# A Comprehensive Review of the Classification, Sources, Phytochemistry, and Pharmacology of Norditerpenes

**DOI:** 10.3390/molecules29010060

**Published:** 2023-12-21

**Authors:** Ni Zeng, Qiongdan Zhang, Qingying Yao, Gang Fu, Wei Su, Wei Wang, Bin Li

**Affiliations:** TCM and Ethnomedicine Innovation & Development International Laboratory, School of Pharmacy, Hunan University of Chinese Medicine, Changsha 410208, China; 20213631@stu.hnucm.edu.cn (N.Z.); 17873338539@163.com (Q.Z.); qingyingyao0616@163.com (Q.Y.); fugang@stu.hnucm.edu.cn (G.F.); suwei0310@163.com (W.S.)

**Keywords:** norditerpenes, norditerpenoids, chemical constituents, biological activities, antimicrobial, anti-inflammatory

## Abstract

Norditerpenes are considered to be a common and widely studied class of bioactive compounds in plants, exhibiting a wide array of complex and diverse structural types and originating from various sources. Based on the number of carbons, norditerpenes can be categorized into C19, C18, C17, and C16 compounds. Up to now, 557 norditerpenes and their derivatives have been found in studies published between 2010 and 2023, distributed in 51 families and 132 species, with the largest number in Lamiaceae, Euphorbiaceae, and Cephalotaxaceae. These norditerpenes display versatile biological activities, including anti-tumor, anti-inflammatory, antimicrobial, and antioxidant properties, as well as inhibitory effects against HIV and *α*-glucosidase, and can be considered as an important source of treatment for a variety of diseases that had a high commercial value. This review provides a comprehensive summary of the plant sources, chemical structures, and biological activities of norditerpenes derived from natural sources, serving as a valuable reference for further research development and application in this field.

## 1. Introduction

Diterpenes are natural terpenes composed of twenty carbon atoms in their molecules and are formed by the polymerization of four isoprene units. They are widely distributed in plants, particularly in plant-secreted milk and resin. In addition to plants, diterpenes can also be found in fungal metabolites and marine organisms. Generally consisting of 20 carbons, diterpenes can give rise to norditerpenes with fewer carbon atoms due to the absence of one to three carbon atoms within the diterpene core structure. C19 norditerpenes, which lack one carbon atom, represent the most common structural type among norditerpenes. Natural sources of norditerpenes exhibit diverse pharmacological activities including anti-inflammatory, anti-tumor, and antimicrobial effects. Therefore, extensive attention has been drawn toward research on norditerpenes. Norditerpene alkaloids are a class of norditerpenes. Yong Shen and coworkers reviewed 337 naturally occurring diterpene alkaloids, including 251 norditerpene alkaloids, derived from studies published between 2008 and 2018 [1]. To avoid the duplication of previous work, this article reviews the structural types and biological activities of norditerpenes (norditerpene alkaloids are not included) derived from studies published between 2010 and 2023. In general, 557 norditerpenes and their derivatives were found in the natural world, distributed into 132 species and 51 families (Table 1). This provides the basis for further research on the discovery of natural product drugs.

## 2. Chemical Constituents of C19 Norditerpenes

**Figure 1:**
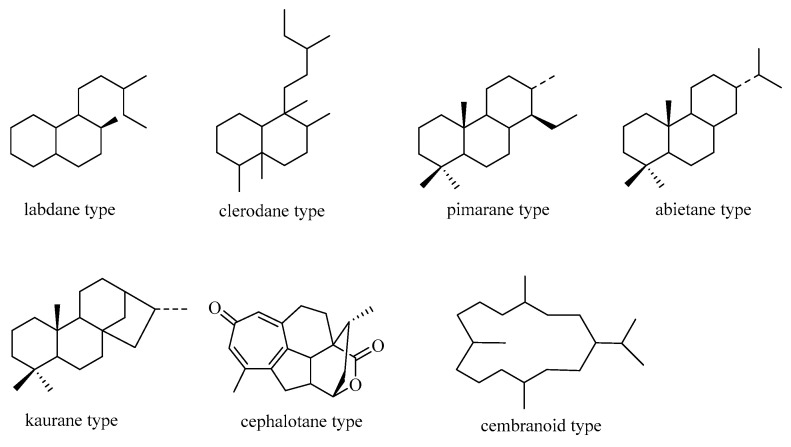
Basic skeleton of C19 norditerpenes.

In nature, seven primary categories of C19 norditerpenes have been identified: the labdane, pimarane, abietane, kaurane, clerodane, cephalotane, and cembranoid types. These types are shown in Figure 1 as their fundamental frameworks. Additionally, there are other norditerpenes with distinctive structures in the natural environment.

### 2.1. Labdane

**Figure 2:**
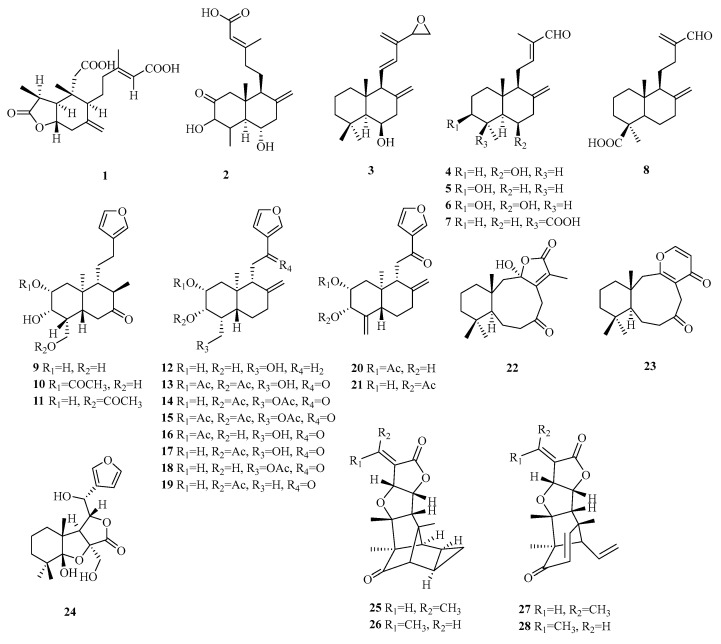
Structures of labdane-type C19 norditerpenes.

Labdane norditerpenes are commonly found as bicyclic diterpenes, with a trans-fused A/B ring in the nuclear parent and a side chain typically consisting of a six-carbon open chain. The presence of hydroxyl groups on the side chain allows for easy dehydration and condensation reactions, leading to the formation of a five-membered ring. Labdane-type diterpenes with both an open-chain structure and a five-membered ring in the side chain have a wide distribution (Table 2, Figure 2).

Compound **1** is a novel diterpene belonging to the 3-*nor*-2,3-*seco*-labdane class. It is characterized by fission the between C-2 and C-3 of ring A, followed by decarboxylation at C-3. Compound **2** is a rare 19-*nor* labdane-type diterpene isolated from fungus, while compounds **3**–**8** possess a fused bicyclic fragment with a 6/6 fusion, both of which are classified as 15-norditerpenes. Compounds **9**–**21** belong to the *ent*-*nor*-furano diterpene of the labdane series. Compounds **22**–**23** feature unique nine-membered ring structures, showcasing unprecedented characteristics. Compound **23** exhibits an exceptional structural motif with a fused *α*, *β*-unsaturated-*γ*-lactone unit, and a nine-membered ring B at C-10/C-11. Compound **24** represents a rare instance of a 6-norlabdane-type diterpene containing a tetrahydrofuran-lactone moiety. Compounds **25**–**26** display unparalleled hexacyclic structures as 19-*nor*-secolabdane diterpenes with tetracyclodecane skeletons. Compounds **27**–**28** are structurally similar to compounds **25**–**26** and exist as *Z*(*E*)-isomers.

### 2.2. Clerodane

**Figure 3:**
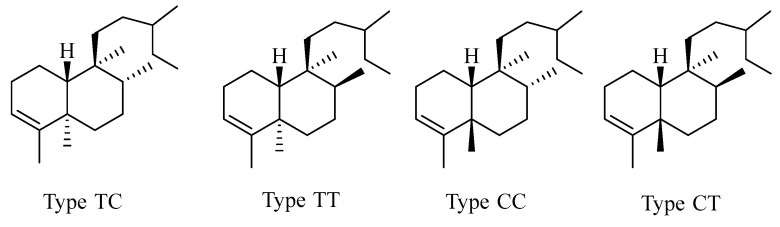
Basic skeletons of clerodane-type C19 norditerpenes.

Clerodane norditerpenes consist of a fused-ring decalin moiety (C_1_–C_10_) and a six-carbon side chain at C-9. Based on the A/B ring junction configuration and the substituents on C-8 and C-9, its skeletons can be classified into four types (Figure 3).

**Figure 4:**
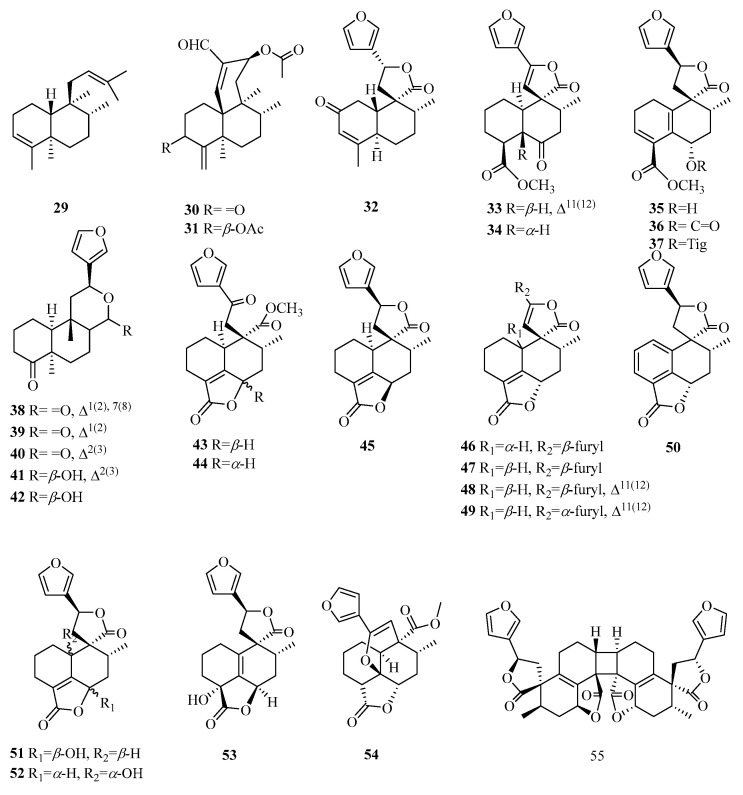
Structures of clerodane-type C19 norditerpenes.

Compound **29** is characterized by a double bond between carbon atoms C-3 and C-4. Compounds **30**–**31** have a fused tricyclic ring system with a 6/6/6 configuration. Compound **32** belongs to the bioactive clerodane class of 19-*nor*-diterpenes, featuring a cyclohexenone decalin ring. Compound **33** is a dehydrogenated derivative of compound **34**, resulting in the removal of hydrogen atoms from positions C_11_–C_12_. Compounds **35**–**37** exhibit a distinct 3,5(10)-diene moiety. Compounds **38**–**40** belong to the furanoditerpenes of the 18-*nor*-clerodane class. Compounds **43**–**44** display an opened lactone ring at positions 17 and 12, accompanied by oxidation occurring at position C-12. Compounds **43**–**52** possess a butenolide moiety extending from C-19 to C-6. Compound **53** demonstrates a similar structure, albeit featuring a double bond between C-5 and C-10. Compound **54** consists of a unique cage-like tetracyclic ring system, fused in a pattern of 6/6/6/5, which is formed by the incorporation of a 5,12-epoxy ring. Compound **55** is identified as an exceptionally symmetrical diterpene dimer characterized by the formation of a cyclobutane ring through [2 + 2] cycloaddition (Table 3, Figure 4).

### 2.3. Pimarane

**Figure 5:**
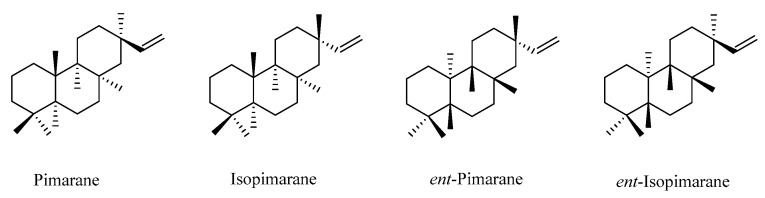
Basic skeletons of pimarane-type C19 norditerpenes.

**Figure 6:**
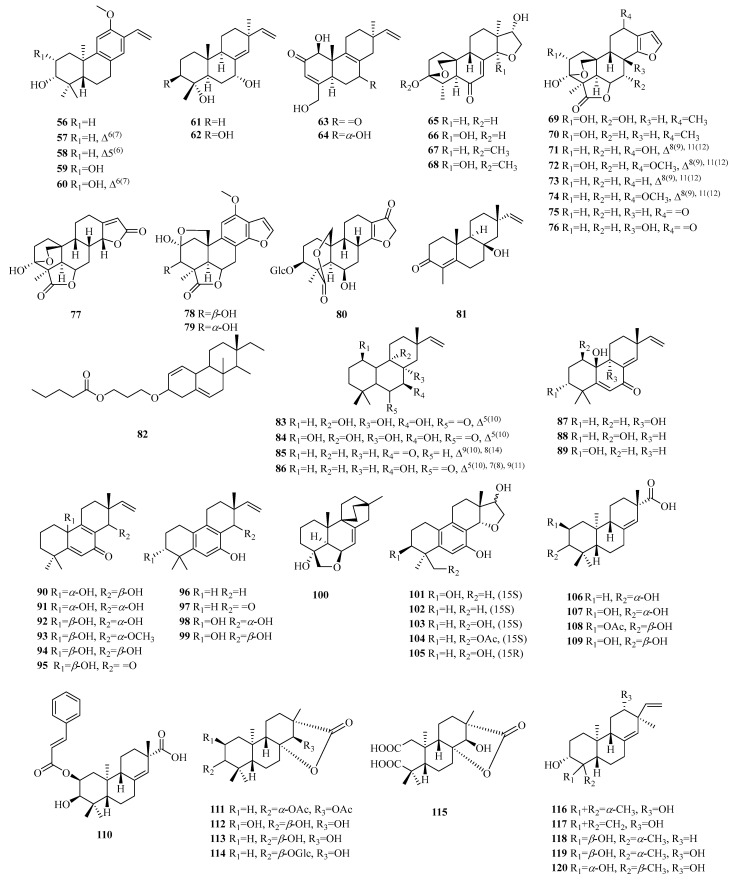
Structures of pimarane-type C19 norditerpenes.

Pimarane norditerpenes are tricyclic diterpenes and are widely distributed in nature. Pimarane norditerpenes are mainly divided into four types (Figure 5): pimarane, isopimarane, *ent*-pimarane, and *ent*-isopimarane. Isopimarane norditerpenes are a class of compounds classified by pimarane norditerpenes according to the difference of chiral centers in the molecule, and they are also the largest number of pimarane norditerpenes (Table 4, Figure 6).

#### 2.3.1. Pimarane

Compounds **56**–**75** belong to the category of pimarane-type norditerpene compounds. Compounds **56**–**60** featured a methoxy group at C-12 and included a vinyl fragment at C-13. Compounds **63**–**64** are exceptional members within the bacterial norditerpene metabolite family. Compounds **65**–**77** exhibit a tetracyclic structure with a 3,20-epoxy bridge, except for compounds **69**–**77**, which also feature lactone carbon at C-19, indicating the presence of the 19,6-gamma-lactone moiety. Compound **77** has been identified as (9*β*H)-17-norpimarane dilactone. Additionally, both compounds **78** and **79** exhibit *β* orientation of the 2,20-epoxy bridge. Compound **80** represents the first example of a glucoside derivative of 17-nor-pimarane diterpenes.

#### 2.3.2. Isopimarane

Compounds **81**–**105** are isopimarane-type norditerpene compounds, with compound **82** featuring a bulky *O*-propyl pentanoate group at C-3. Compounds **90**–**95** exhibit unique examples of the 20-*nor*-isopimarane type, characterized by the presence of a cyclohexa-2,5-dien-1-one moiety. Compounds **96**–**99** represent four aromatic norditerpenes. Compound **100** possesses an unusual skeleton composed of an 18-*nor*-9, 16-cyclo-isopimarane framework with a cage-like bicycle [2.2.2] octane moiety. Lastly, compounds **101**–**105** display a rare 14,16-cyclic ether unit and possess a unique 6/6/6/5 tetracyclic cycloether skeleton.

#### 2.3.3. Ent-Pimarane

Compounds **106**–**109** exhibit an ester bond instead of the olefinic bond at C-13. Compound **110** displays an additional cinnamoyl group and a vinyl benzene moiety. Compounds **111**–**115** are characterized by a rare 16-*nor*-*ent*-pimarane skeleton and demonstrate the presence of *γ*-lactone between the C_15_-O-C_8_ bonds. Compound **115** represents the first reported instance of this previously undescribed skeleton of 2, 3-*seco*-16-*nor*-*ent*-pimarane.

#### 2.3.4. Ent-Isopimarane

Compounds **116**–**117** represent the first examples of 18 (or 19)-*ent*-isopimarane norditerpenes. Compound **117** has a double bond at C-14, while compounds **118**–**120** feature a hydroxyl group at C-4, with compound **119** being the C-4 epimer of **120**.

### 2.4. Abietane

**Figure 7:**
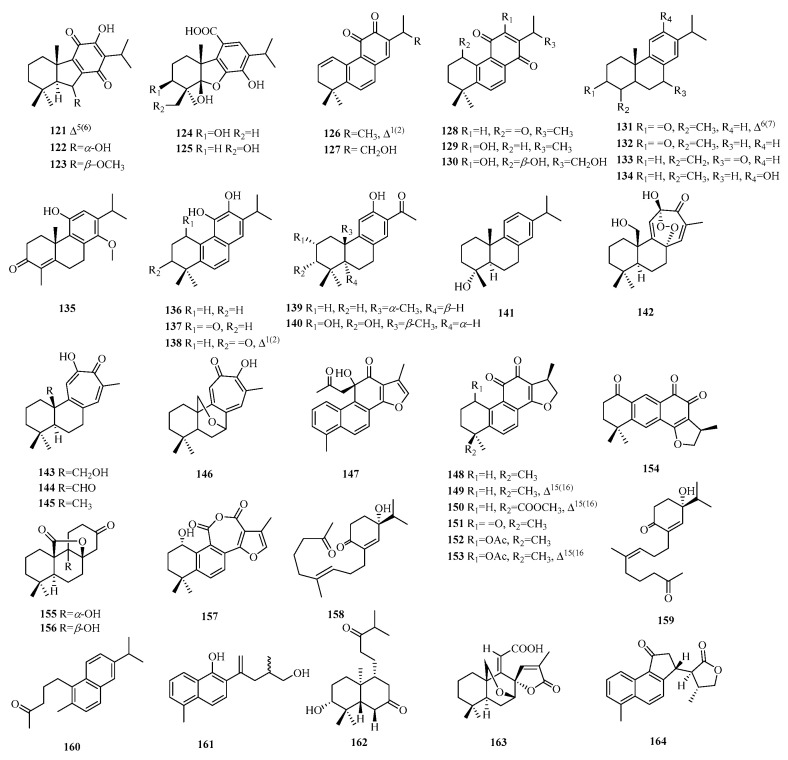
Structures of abietane-type C19 norditerpenes.

Abietane norditerpenes are tricyclic diterpenes commonly formed through rearrangements of pimarane norditerpenes. The core structure consists of a hydrogenated phenanthrene with an isopropyl group at C-13, geminal dimethyl groups at C-4, and a methyl group at C-20. Ring B and the side chains on the C ring can easily undergo rearrangement to form a five-membered ring, with some quinone structures also found on the C ring. Abietane norditerpenes are primarily classified into abietane type, *ent*-abietane type, and *seco-*abietane type (in Table 5, Figure 7).

Compounds **121**–**123** belong to the 8-*nor*-7(8→14),9(8→7)-di-abeo-abietane skeleton type. Compounds **124**–**125** possess a 6-*nor*-6,7-*seco*-abietane skeleton. Compounds **126**–**127** contain a moiety of 1,2-quinone, while compounds **128**–**130** feature a moiety of 1,4-quinone in ring C. Compounds **126**–**141** are aromatic norditerpenes, while compounds **142**–**146** feature a tropolone moiety; notably, compound **142** is characterized by the presence of a peroxide group connecting C-8 and C-12. Furthermore, compounds **147**–**153** demonstrate rearranged side chains that form an oxygen-containing five-membered ring. Compounds **155**–**156** possess a unique *γ*-lactone subgroup located between C-8 and C-20. Compound **157** is a seven-membered norditerpene featuring two carbonyl groups in the C ring. Compounds **158**–**162** are *seco*-abietane norditerpenes. Compounds **158**–**159** possess a unique 18-*nor*-5,10: 9,10-disecoabietane skeleton, while compound **160** exhibits a rearranged angular methyl group at C-5 and belongs to the 4, 5-*seco*-19-*nor*-abietane skeleton. Compound **163** is characterized by a 7*β*,19-epoxydecalin spirally linked with a five-membered *α*, *β*-unsaturated lactone ring, and an acrylic acid moiety.

### 2.5. Kaurane

**Figure 8:**
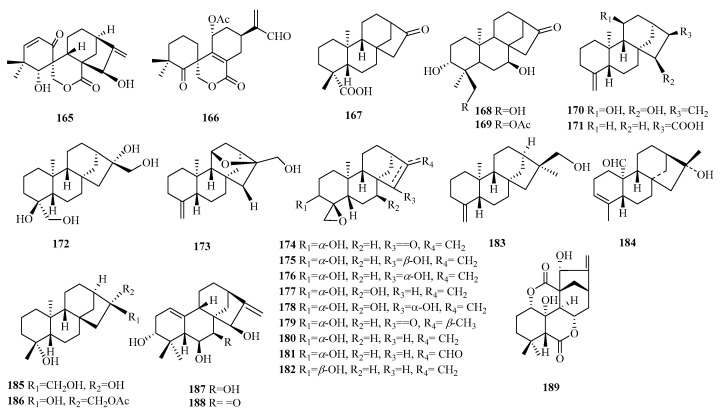
Structures of kaurane-type C19 norditerpenes.

Kaurane norditerpenes represent a class of tetracyclic diterpenes characterized by the hydrophenanthrene core skeleton. These compounds can be classified into two configurations, namely kaurane and *ent*-kaurane. It is worth noting that the ent-kaurane-type norditerpenes exhibit remarkable abundance (Table 6, Figure 8).

Compounds **165**–**166** are spiral seco *ent*-kaurane norditerpenes. Compound **165** is a naturally occurring compound with a structure of 6-*nor*-6,7-*seco*-*ent*-kauranoid, while compound **166** featured a 6,7: 8,15-*seco*-*ent*-kaurane diterpene skeleton. 

Compounds **170**–**182** are classified as *ent*-18-norkaurene-type diterpenes. Compound **173** features an 11*β*,16*β*-epoxy ring moiety. Compounds **174**–**182** exhibit a 4*β*, 19-epoxy ring moiety. Compounds **183**–**186** are categorized as C-19 *nor*-*ent*-kaurane norditerpenes. 

Compounds **187**–**188** demonstrate a unique structural pattern of 20-*nor*-*ent*-kaurane norditerpenes. Compound **189** is determined to be a *nor*-6,7-*seco*-1,7: 6, 11-diolide-*ent*-kaurane, and the first diterpenene within the category of the 20-nor-enmein type.

### 2.6. Cephalotane

**Figure 9:**
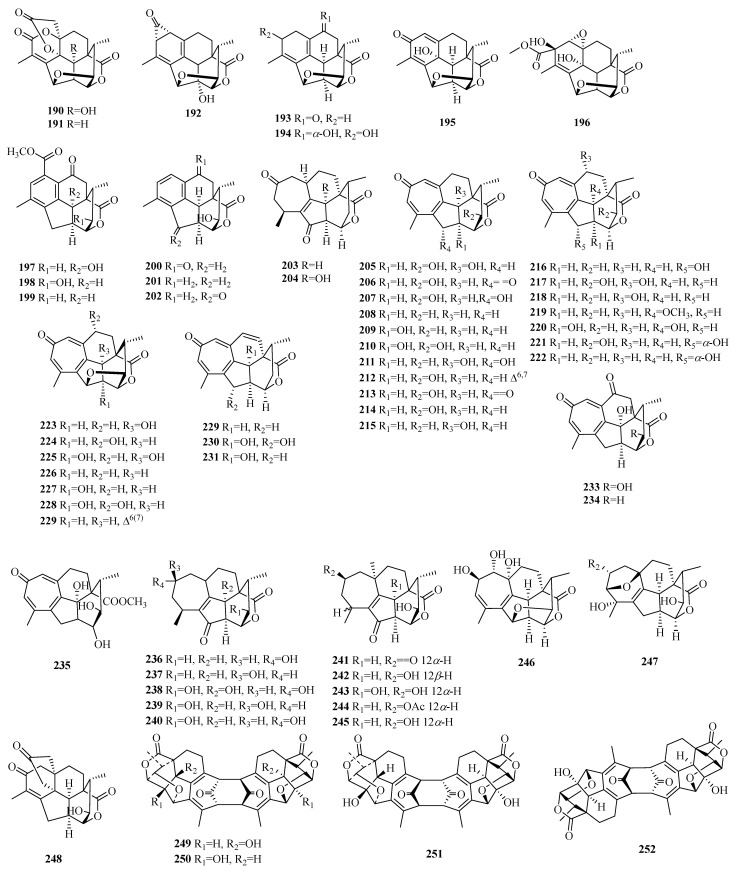
Structures of cephalotane-type C19 norditerpenes.

In the past decade, more than fifty cephalotane-type norditerpenes have been discovered through previous phytochemical investigations, which can be categorized into four distinct structural groups: A-ring-contracted cephalotane-type norditerpenes, cephalotaxus troponoids (C19), 17-*nor*-cephalotane-type diterpenes (C19), and cephalotane dimers (Table 7, Figure 9).

Compounds **190**–**202** are norditerpenes featuring a contracted A-ring structure. Compound **192** exhibits a distinctive bicyclo [4.1.0] hepta-2,4-dien-7-one moiety. Compounds **203**–**235** belong to cephalotaxus troponoids, which represent the predominant group of cephalotane-type norditerpenes. The skeletal structure of cephalotaxus troponoids is distinguished by a highly rigid tetracyclic carbon framework, encompassing a tropone moiety, multiple oxygenated groups, and methyl substituents at positions C-4 and C-12. Cephalotaxus troponoids are derived from labdane-type norditerpenes. The 17-*nor*-cephalotane-type diterpenes were derived from cephalotaxus troponoids via a reduction in the tropone ring. Compounds **236**–**248** exemplify this class of diterpenes, while compound **247** features an 8-oxabicyclo [3.2.1] oct-2-ene moiety. Compounds **249**–**252** represent the first example of C19 norditerpene dimers and possess a unique tricycle [6.4.1.1^2, 7^] tetradeca-3,5,9,11-tetraene-13,14-dione core, and both ends are terminated with a C_2_ symmetric or asymmetric rigid polycyclic ring system.

### 2.7. Cembranoid

**Figure 10:**
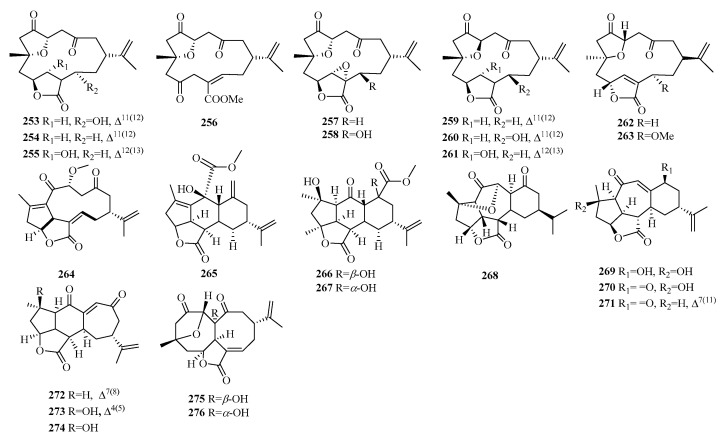
Structures of cembranoid-type C19 norditerpenes.

Cembranoid-type norditerpenes are macrocyclic diterpenes that belong to a class of natural products characterized by fourteen-membered rings and possess three symmetrically distributed methyl groups and one isopropyl group. Isopropyl-cembrane-type norditerpenes, primarily formed through isopropyl or isopropenyl substitution, represent the most common cembrane-type norditerpenes (Table 8, Figure 10).

Compounds **253**–**263** are characterized by the presence of polycyclic furanobutenolide-derived norcembranoid diterpenes isolated from soft corals. Compound **264** possesses a rare 5/5/11-fused tricyclic ring system, while compounds **265**–**267** possess a 5/5/6/6 tetracyclic ring system. Compounds **268**–**274** possess a cyclopentane ring, a cyclohexane ring, and a seven-membered ring. Compounds **275**–**276** display an uncommon 8/8 bicyclic carbon core.

### 2.8. Others

Compounds **277**–**278** are lathyrane-type norditerpenes. Compound **279** is an atypical C19 furano-norditerpene. Compounds **280**–**282** have uncommon characteristics as cassane-type norditerpenes. The classification of compounds **283**–**286** confirms their identity as cleistanthane-type norditerpenes. The discovery of compounds **283**–**284** marks the first examples of phenylethylene-bearing derivatives among 20-nor-diterpenes, with compound **284** exhibiting a unique 3,10-oxybridge moiety. Compound **286** stands out due to its scarcity among phenylacetylene-containing 18-nor-diterpene glycosides. Compounds **287**–**291** exhibit the characteristic fusicoccane-type norditerpenes. Specifically, compounds **287**–**289** belong to a unique category of 16-nor-dicyclopenta [a,d]-cyclooctane norditerpenes, while compound **287** features an undescribed tetracyclic ring system with a configuration of 5/6/6/5. Compounds **292**–**293** are obtained through the rearrangement of the crotofolane skeleton. Compounds **294**–**295** exhibit a pair of enantiomeric norditerpenes featuring an unexpected 6/5/6/6-fused tetracyclic ring system. Compounds **296**–**298** are uniformly classified as ent-atisane norditerpenes. Compound **299** possesses a perhydroazulene ring system, while compounds **300**–**302** represent three newly rearranged oxygenated terpenes. Compounds **303**–**304** belong to the class of spongian diterpenes and exhibit a highly distinctive carbon skeleton known as 3-nor-spongian. Li’s group unveiled four unprecedented C19 norditerpenes, including compound **305** with a cyclopenta[b]furan-2,5-dione skeleton, compound **306** with a pyran[b]furan-2,6-dione skeleton, and compounds **307**–**308**, which are epimerides possessing a pair of dioxaspiro[4.4]nonane skeletons.

**Figure 11:**
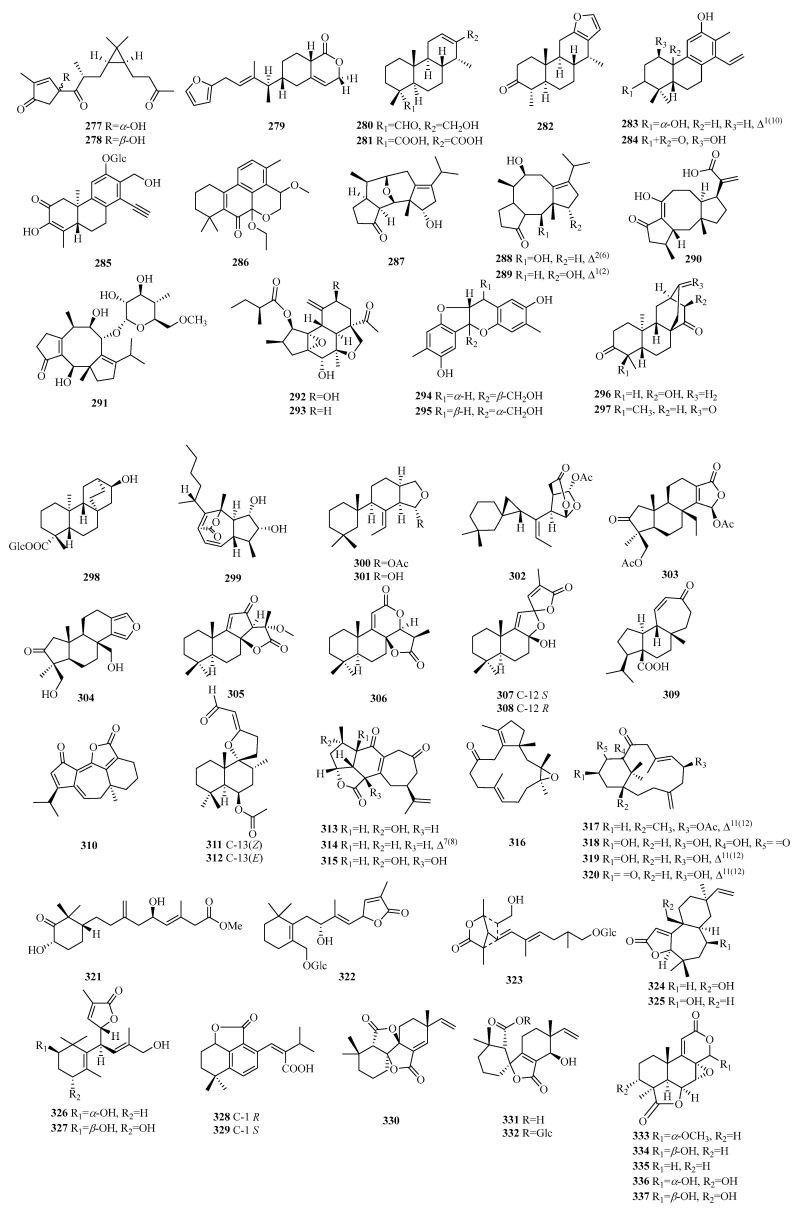

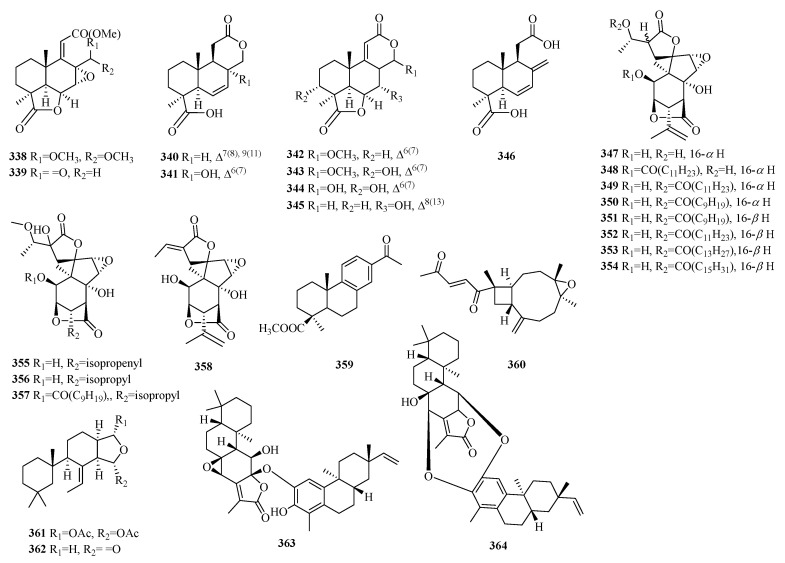
Structures of other compounds of C19 norditerpenes.

Compound **309** is a naturally occurring norditerpene with a seven-membered ring mulinane skeleton lacking the C-16-methyl group. Compound **310** possesses an undescribed nor-guanacastane skeleton. Compound **312** has been identified as an isomer of **311**. Compounds **313**–**314** belong to yonarane norditerpenes, and compound **317** belongs to inelegane-type norditerpenes. Compound **315** is considered an undescribed bicyclo[11.3.0]hexadecane carbon skeleton, whereas compounds **316**–**321** are consistently classified as verticillane-type norditerpenes. Compounds **322**–**323** are norditerpene glycosides. Compounds **324**–**332** represent new norditerpene lactones, whereas compounds **333**–**345** are norditerpene oidiolactones. Compounds **347**–**358** have been identified as norditerpene picrotoxanes. Compound **359** exhibits a podocarpane skeleton, while compound **360** belongs to the xeniaphyllane-type norditerpene. Compounds **363**–**364** are a heterodimeric diterpene consisting of an ent-abietane and an 18-nor-rosane skeleton with an aromatic ring (Table 9, Figure 11).

## 3. Chemical Constituents of C18 Norditerpenes

**Figure 12:**
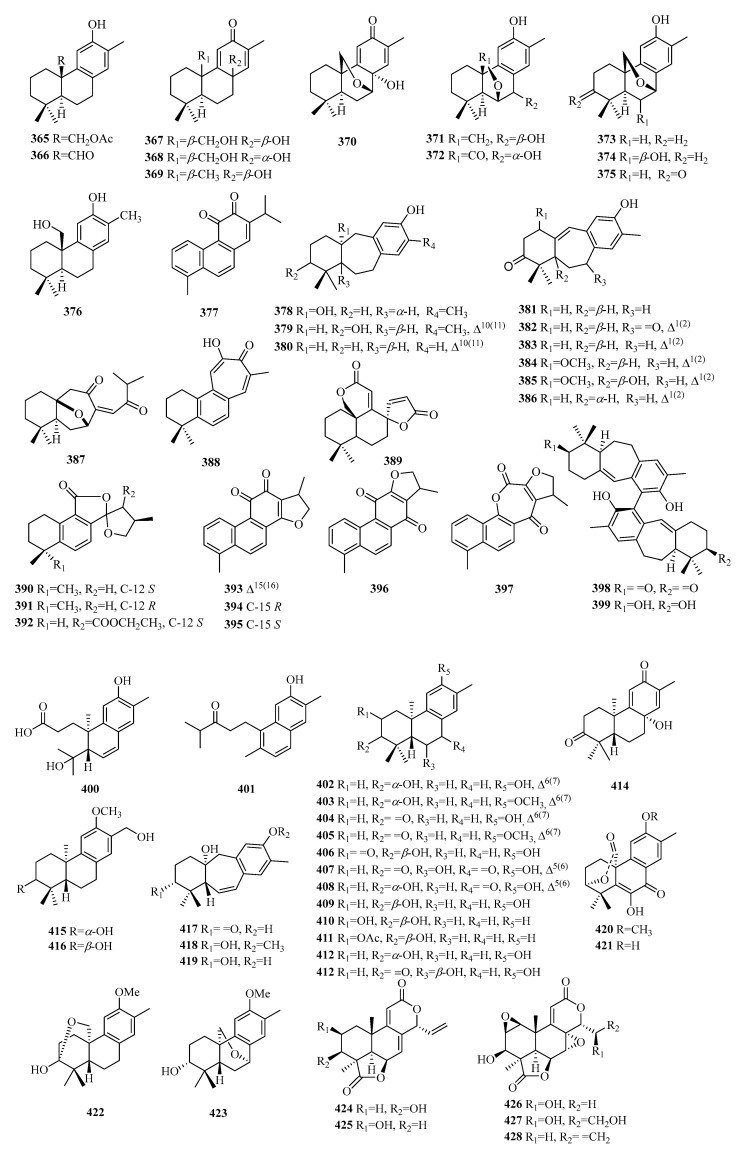

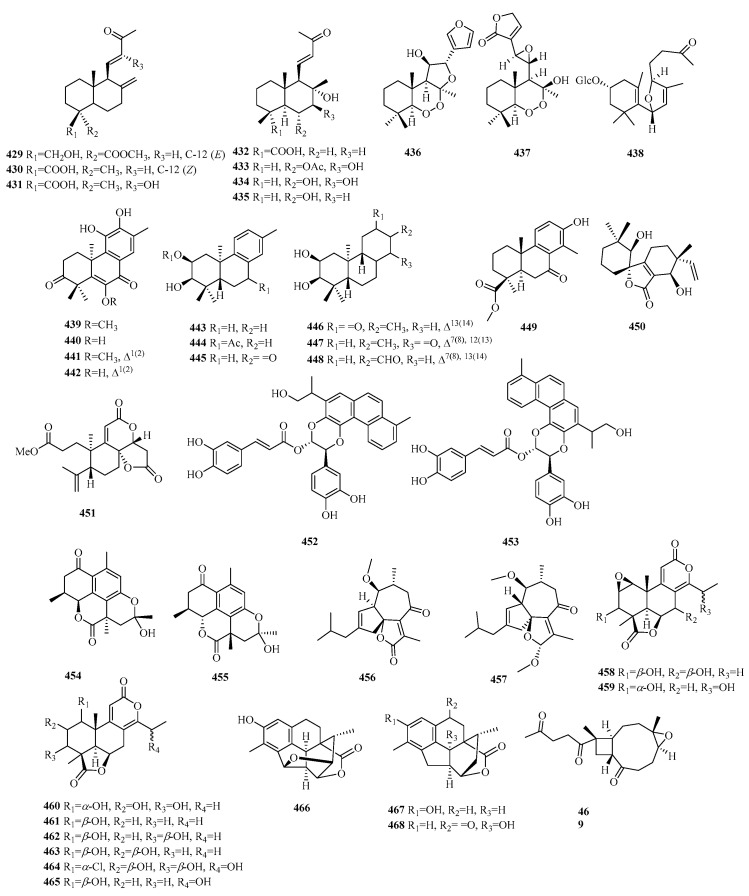
Structures of C18 norditerpenes.

Ten primary categories of C18 norditerpenes have been identified in the natural environment, including the labdane, abietane, podocarpane, pimarane, cassane, aspergilane, benzodioxane, commiphorane, totarane, and cephalotane types. Additionally, there are other norditerpenes with atypical structures and certain abietane dipolymers found in nature (Table 10, Figure 12).

### 3.1. Abietane

Compound **365** is a new abietane type and has been reported as a 20-acetyl derivative of 15,16-dinorpymara-8,11,13-trien-12-ol, known as salyunnanin F. Compound **368** is a diastereomer of **367**. Compounds **370**–**375** exhibit an ether bridge between C-18 and C-6 or C-7. Compounds **378**–**388** are dinorditerpenes derived from an abietane-type skeleton with a seven-membered ring in the tricyclic skeleton. Compound **387** is a unique C18 norditerpene bearing a special *seco*-ring C. Compounds **389**–**397** belong to a special class of tetracyclic abietane-type dinorditerpenes. The stem bark of *Trigonostemon chinensis* yielded two novel dimeric degraded diterpenes, compounds **398**–**399**, featuring a homodimeric biaryl skeleton derived from the rearrangement of chiral nonracemic abietane-type norditerpenes. This structural motif is connected through an axially chiral biaryl 11,11′-linkage.

### 3.2. Podocarpane

The podocarpane type is a trinucleated norditerpene. Compounds **400**–**414** and **420**–**423** are new 13-methyl-*ent*-podocarpane norditetrpenoids, while **427**–**428** are 12-methyl-*ent*-podocarpane norditetrpenoids.

Compounds **400**–**401** exhibit a unique dinorditerpene bearing a special *seco*-ring A, and **400** is a 13-methyl-3,4-*seco*-*ent*-podocarpane norditetrpenoid. Compounds **417**–**419** are classified as 13-methyl-9(10→20)-abeo-*ent*-podocarpane norditetrpenoids. Compounds **420**–**431** are 13-methyl-7-oxo-*ent*-podocarpane norditetrpenoids, and compound **420** is a derivative methylated at position 12 of compound **421**. Compounds **424**–**428** belong to a special class of tetracyclic diterpenes.

### 3.3. Other Compounds

Compound **429** is an oxidized derivative of the common C20 labdane precursor and is identified as a 15,16-dinor labdane diterpene. Compounds **430**–**435** are determined to be 14,15-*bisnor* labdane diterpenes. Compounds **436**–**437** present the 6,7-dinorlabdane diterpenes with a peroxide bridge. Compound **438** is a special norditerpene glucoside and is defined as lyonivaloside I.

Compounds **439**–**448** contain a unique skeleton of 15,16-*dinor*-*ent*-pimarane diterpenes. Compound **449** represents a novel phenolic cassane norditerpene. Compound **450** exhibits a new aspergilane skeleton in structure with a special 6/5/6 tricyclic system containing an *α*,*β*-unsaturated spironolactone moiety in the B ring. Compound **451** is a novel natural product with a rare 6/6/5-fused tricyclic ring system. Compounds **452**–**453** are two compounds consisting of dinorditerpene and 90-norrosmarinic acid derivatives and are linked by a 1,4-benzodioxanyl motif. Compounds **454**–**455** are aromatic tetranuclear terpenoids with unprecedented carbon skeletons from *Resina Commiphora* and possess an uncommon 6/6/6/6 ring system. Compounds **458**–**465** have been identified as a series of totarane-type norditerpenes. Compounds **464**–**468** exhibit the rare characteristic of A-ring contraction, known as cephanolide A–C. Compound **469** possesses a skeleton of 17,19-dinorxeniaphyllane.

## 4. Chemical Constituents of C17 Norditerpenes

**Figure 13:**
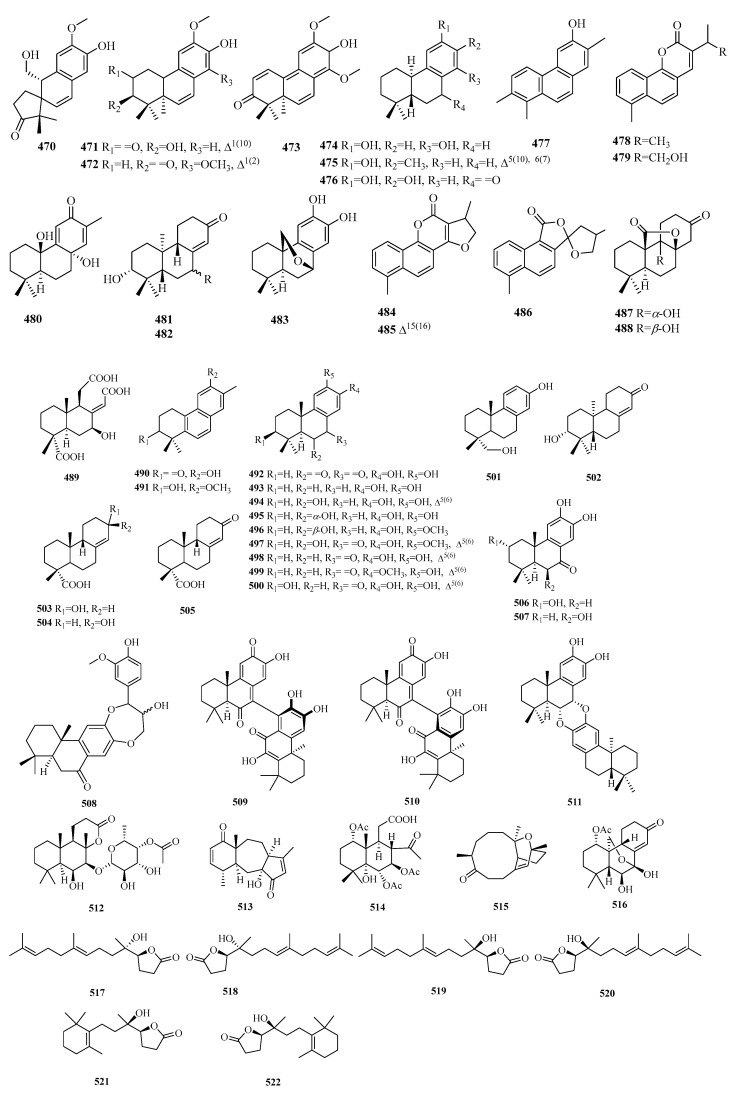
Structures of C17 norditerpenes.

Seven major types of C17 norditerpenes have been reported in nature: the labdane type, abietane type, podocarpane type, briarane type, cassane type, cembrane type, and kaurane type. Additionally, there are other trinorditerpenes with special structures and some podocarpane dimers in nature (Table 11, Figure 13).

### 4.1. Abietane

Compounds **470**–**477** are 15,16,17-bis-norditerpenes. Compound **479** is the 16-OH derivative of **478**. Compound **480** represents a rare skeleton of a 20-nor-abietane. Compound **482** is defined as the C-7 epimer of compound **485**, while compounds **484**–**486** are aromatic tetranuclear terpenoids with unprecedented carbon skeletons from *S. digitaloides*. Compounds **487**–**488** possess a unique γ-lactone subunit moiety positioned between C-8 and C-20, leading to the generation of the carbonyl carbon at C-13 through the degradation of the isopropyl group.

### 4.2. Podocarpane

Compounds **489**–**511** are trinorditerpenes of the podocarpane type. Compounds **509**–**510**, existing as atropisomers, are two previously unknown dimeric trinorditerpenes separated from the root bark of *C. orbiculatus*. Compound **511** is a rare dimeric trinorditerpene with a 1,4-benzodioxane moiety.

### 4.3. Others

Compound **512** is a novel labdane-norditerpenoid glycoside with a six-membered epoxy system (8→13). Compound **513** is a novel norditerpene derived from a briarane skeleton. Compound **514** represents a previously unidentified trinorcassane diterpenoid bearing a dicyclic norditerpenoid with three acetoxy moieties. Compound **515** is determined to be a cembrane-type macrocyclic trinorditerpenoid with a fused 10/6 carbon skeleton. Compound **516** is a unique trinorditepenoid containing an unprecedented 20-epoxy-ent-kaurane skeleton. Compounds **517**–**521** are three pairs of unfrequent C17 γ-lactone norditerpenoid enantiomers.

## 5. Chemical Constituents of C16 Norditerpenes

**Figure 14:**
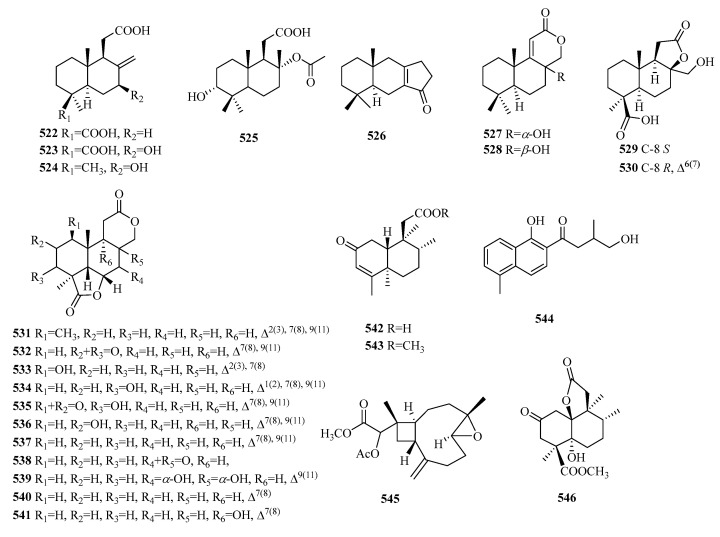

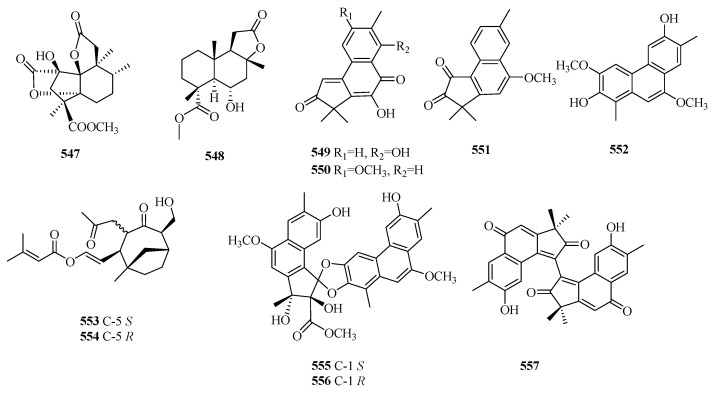
Structures of C16 norditerpenes.

Four main types of C16 norditerpenes have been found in nature: the labdane type, clerodane type, xeniaphyllane type, and abietane type. In addition to the common four types, there are also other norditerpenoids with unique structures and some dimers in nature sources (Table 12, Figure 14).

### 5.1. Labdane

C16-labdane norditerpenes are common bicyclic diterpenes with four fewer carbon atoms. Compounds **522**–**525** are known as bicyclic norditerpenoids. Compound **525** is the first to be reported in nature. Compound **526** is a rare rearranged labdane-type tetranorditerpenoid with a fused tricarbocyclic system (6/6/5) and an α,*β*-unsaturated cyclopentenone unit in ring C. Compounds **527** and **528** represent a pair of new labdane-type tetranorditerpenoid epimers, which are the first known examples of naturally derived labdane tetranorditerpenoids. Compounds **531**–**541** are a series of tetracyclic tetranorlabdane diterpenoids.

### 5.2. Others

Compounds **542**–**543** exhibit clerodane-type tetranorditerpenoids. Compound **544** is a novel abietane tetranorditerpenoid, known as castanol C. Compound **545** possesses an unprecedented carbon skeleton derived from xeniaphyllane. Compound **546** is a newly reported tetranorditerpenoid featuring a special fused ring system of 6/6/5. Compound **547** is a new C16 tetranorditerpenoid lactone with an uncommon tetracyclic fuse system of 5/5/5/6. Compounds **549**–**551** possess an unprecedented framework of 2H-benz[e]inden-2-one. Compounds **552**–**553** represent a pair of epimers of vibsane-type tetranorditerpenes with a bicyclo[4.2.1]nonane unit. Compounds **555**–**556** comprise a pair of rearranged tetranorditerpenoid dimers with a spiroketal core moiety.

## 6. Pharmacological Activities

Norditerpene, a substance of profound pharmacological significance, manifests diverse primary pharmacological effects and biological activities encompassing cytotoxicity, anti-inflammatory activity, and antibacterial and antiviral actions, as well as antioxidant potential.

### 6.1. Cytotoxic activity

The anti-tumor activity of numerous norditerpenes has been extensively reported. The cytotoxicity of norditerpenes is the most frequently studied biological activity, and a table listing their cytotoxic activities is provided (Table 13). The cytotoxic activity of compound **208** was found to be the most potent against A549, Hela, and SGC-7901 cell lines, with IC_50_ values of 0.10 µM, 0.13 µM, and 0.14 µM, respectively [78].

### 6.2. Antimicrobial Activity

#### 6.2.1. Antibacterial Activity

Inulifolinone D (**16**) from *A. inulifolium* can enhance the bactericidal activity against *S. aureus*. (MIC = 150–75 μg/mL) [10]. Actinomadurol (**64**) showed high antibacterial activity against pathogenic strains, including *Staphylococcus aureus*, *Rhizophila Kocuria,* and *Proteus Hauseri*, with MICs ranging from 0.39 to 0.78 μg/mL. However, Jbir-65 (**63**) did not exhibit any antibacterial activity, suggesting that the hydroxyl group at C-7 was responsible for antibacterial activity [25]. Compounds **69**–**72**, **74**, and **79**–**80** from *I. Trichantha* showed antibacterial activity against both standard and resistant strains of *H. pylori*, with an MIC of 8–64 μg/mL. In addition, compounds **72** and **74** showed superior antibacterial activity against *H. pylori* compared to other compounds, with MIC values ranging from 8 to 16 μg/mL. SAR studies have demonstrated that CH_3_O-12 and 3, 20-epoxy moiety in 17-*nor*-pimarane diterpenes can function as activating groups, while the sugar moiety at C-2 might an inactivated group. The combination of antibiotic-icacinlactone B with either metronidazole or amoxicillin exhibited notable additive effects (FICI = 0.56–0.75) against the clinical strain HP159, suggesting that combination therapy has the potential to enhance antimicrobial activity, reduce dosage requirements, and mitigate adverse side effects. Compounds **87**, **89**, **96**, **98**, and **99** displayed inhibitory activities against *E. tarda*, *M. luteus*, *P. aeruginosa*, *V. harveyi*, and *V. parahemolyticus*, with MIC values of 4.0 μg/mL each; compounds **87** and **99** also exhibited activity against the *F. graminearum,* with MIC values of 2.0 and 4.0 μg/mL, respectively [34]. Compounds **90**–**91** exhibited comparable MIC values of 8.0 μg/mL against zoonotic pathogenic bacteria, including *E. coli*, *E. tarda*, *V. harveyi*, and *V. parahaemolyticus*. Compound **93** exhibited potent activity (MIC = 4.0 μg/mL) against the plant pathogen *F. graminearum* [35]. Salprzelactone (**164**) showed higher antibacterial activity against *A. aerogenes* than streptomycin, acheomycin, and ampicillin, as indicated by MIC values of 62.5 μg/mL versus 125 μg/mL, 250 μg/mL, and 125 μg/mL, respectively [55]. The growth of *S. aureus* was inhibited by citrinovirin (**299**) at an MIC of 12.4 μg/mL, while it displayed toxicity toward *A. salina,* with an LC_50_ of 65.6 μg/mL. Moreover, compound **299** exhibited inhibitory effects ranging from 14.1% to 37.2% against *C. Marina*, *H. Akashiwo*, and *P.donghaiense* at 100 μg/mL; however, it stimulated the growth of *S.trochoidea* [101]. Compound **531** displayed potent activity against *E. tarda,* with an MIC value of 16 μg/mL [162,166]. Compounds **555**–**556** showed moderate antimicrobial activities against *S. aureus*, 8^#^MRSA, and 82^#^MRSA, with MIC values from 1.56 to 6.25 μg/mL [173].

#### 6.2.2. Antifungal Activity

Compounds **333**, **335**, and **342** displayed antifungal activity against *C. neoformans* (MIC = 17.5, 12.5, and 10.0 μg/mL, respectively) and *C. albicans* (MIC = 20, 20, and 12.5 μg/mL, respectively) [116]. Compounds **333** and **342** also demonstrated inhibitory effects on the growth of *P. destructans,* with MIC values of 7.5 and 15 μg/mL, respectively [116]. Compound **534** showed moderate antifungal activities against *C. albicans,* with an MIC of 16 μg/mL [162].

#### 6.2.3. Antiviral Activity

The compound eupneria J (**118**) displayed potent anti-HIV-1 activity (IC_50_ = 0.31 μM). These findings suggest that the presence of a *β*-oriented hydroxyl group at C-4 may enhance its anti-HIV-1 activity. Furthermore, it is plausible that the acetoxyl group at C-18, rather than at C-3, contributes to an augmented anti-HIV effect [41]. Compound **276** exhibited antiviral activity against human enterovirus EV71, with an IC50 value of 5.0 μM [87]. Compound **394** exhibited anti-HBV activity by effectively suppressing the secretion of HBsAg and HBeAg, with IC50 values of 0.11 and 0.18 μM, respectively. In addition, it also effectively suppressed HBV DNA replication, with an SI value of 647.2 [56]. Compounds **422**–**423** showed potent anti-HCV activity, with EC50 values of 7.5 and 6.6 μM, respectively [128]. Compounds **490**–**491** demonstrated moderate anti-HCV activity, exhibiting EC50 values of 13.0 ± 0.3 μM and 23.6 ± 1.9 μM, respectively [148].

### 6.3. Anti-Inflammatory

The activation of NLRP3 inflammasome causes pyroptosis and results in the maturation of caspase-1 and the secretion of IL-1*β* [15]. The anti-NLRP3 inflammasome effects of compound **31** were evaluated by MCC950 (IC_50_ = 23.1 ± 5.3 µM) as a positive control, which can specifically inhibit NLRP3 inflammasomes. Compound **31** inhibited IL-1*β* secretion (IC_50_ = 5.5 ± 3.2 µM) and maturation of caspase-1 in a dose–dependent manner, indicating that cell pyroptosis is prevented, thereby demonstrating its ability to inhibit NLRP3 inflammasome activation [15].

Compound **82** derived from *P. malabarica* showed anti-inflammatory (anti-5-LOX) effects, with IC_50_ values of 0.75 mg/mL, more potent than ibuprofen (IC_50_ = 0.93 mg/mL). In vitro, compared with ibuprofen (selectivity index = 0.44), compound **82** exhibits a higher selectivity index (anti-COX-1_IC50_/anti-COX-2_IC50_ = 0.85), indicating fewer side effects. Notably, compound **82** exhibits promising potential against both cyclooxygenase and lipoxygenase [30].

The inhibitory potential of compound **279** against 5-LOX (IC_50_ = 0.92 mg/mL) was found to be higher than ibuprofen (IC50 = 0.96 mg/mL) [89].

Compound **110** showed significant inhibition, with an IC_50_ value of 14.7 ± 1.8 μM, surpassing the potency of PDTC (Pyrrolidinedithiocarbamate, IC_50_ = 26.3 µM), a well-established positive control for an NF-κB inhibitor [39].

Compound **190** from *C. sinensis* exhibited effective inhibition of NF-κB activity (IC_50_ 4.12 ± 0.61 µM) [73]. Salvialba acid (**163**) has been shown to possess anti-inflammatory effects on TNF-α-induced vascular inflammation in HAECs (human aortic endothelial cells) [60].

Compound **208**, obtained from the twigs and leaves of *C. fortune*, dose–dependently inhibited TNF-*α*-induced NF-κB activation with an IC_50_ value of 0.10 μM, which was similar to the inhibitory effect of the positive control MG132 (a proteasome inhibitor, IC_50_ = 0.15 μM). These results suggest that the tropone moiety is important for the cytotoxicity and the inhibition of NF-κB signaling [78].

Compounds **255**, **261**, and **269** also displayed inhibitory effects on NF-κB with inhibitory rates of 28.6%, 25.1%, and 12.5% in a dose of 50 µM compared to the positive control PDTC (IC_50_ = 37 µM) [82,86].

Compounds **494**–**496** and **500** demonstrated moderate inhibitory activities against NF-κB activation in RAW264.7 macrophages, with IC_50_ values of 15, 25, 19, and 4 μM, respectively [149,150,151]. Compound **8** was the most effective compound in inhibiting LPS-induced nitric oxide (NO) production (IC_50_ = 3.56 μM) in *T. orientalis*. Moreover, it attenuated the expression of iNOS and COX-2 at both mRNA and protein levels by inhibiting LPS-induced degradation of I-κBα and activation of NF-κB, as well as reducing ERK phosphorylation [7].

Compounds **324** and **325** exerted pronounced inhibition of NO production in LPS-induced macrophages (RAW 264.7), with IC_50_ values of 7.50 and 6.49 μM, respectively [113].

Compound **437** showed a significant anti-inflammatory effect on LPS-induced NO production in RAW264.7 cells (IC_50_ = 21.0 μM) [12].

Compounds **445**–**448** showed potent inhibitory activity against LPS-induced production of NO and TNF-*α* in RAW264.7 cells, with IC_50_ values of below 25 µM [40,137].

Compounds **373**, **374**, **378**, and **480** exhibited potent inhibition of NO release in the cell culture medium of LPS-stimulated macrophages. Furthermore, they significantly inhibited iNOS expression in J774A.1 macrophages at doses of 50–12.5 μM [122].

Compounds **493** and **508** exhibited potent inhibitory effects on LPS-stimulated NO releases and pro-inflammatory mediators, with IC_50_ values of 4.9 and 12.60 μM, respectively, by suppressing iNOS and COX-2 expressions to prevent NO production [139]. 13-*epi*-scabrolide C (**263**) inhibited the production of IL-12 and IL-6 in LPS-stimulated BMDCs (bone marrow-derived cells, IC_50_ = 5.30 ± 0.21 and 13.12 ± 0.64 μM, respectively). This suggests that the C-13 methoxyl moiety may play an important role in anti-inflammatory activity [82].

Scrodentoids H,I (**307**–**308**) exert anti-inflammatory effects by reducing LPS-induced inflammation and inhibiting the JNK/STAT3 pathway in macrophages. STAT proteins play a pivotal role in modulating cytokine-mediated inflammatory responses, and STAT3 is highly correlated with inflammatory responses. In response to inflammatory stimuli, STAT3 acts as a transcription factor that directly governs the expression of pro-inflammatory cytokines. Scrodentoids H and I might be beneficial in the treatment of inflammatory diseases, like ulcerative colitis and atherosclerotic diseases [105].

Sinusiaetone A (**316**) from *S. siaesensis* exhibited significant inhibition against LPS-induced inflammation in BV-2 microglia at a concentration of 20 μM and also decreased the mRNA levels of pro-inflammatory cytokines IL-6 and IL-1*β* [109].

### 6.4. Antioxidative Activity

Compound **82** showed antioxidant activity comparable to *α*-tocopherol, exhibiting an IC_50_ value of approximately 0.6 mg/mL for DPPH scavenging activity, and it can serve as a natural alternative to synthetic antioxidants [30].

The radical quenching analysis revealed that compound **279** exhibited a higher antioxidant activity (IC_50_ value of 0.60 mg/mL) compared to *α*-tocopherol. This suggests the potential of compound **279** as a natural antioxidant in future applications, attributed to its low hydrophobicity and spatial variability [89].

### 6.5. α-Glucosidase Inhibitory Activity

Compounds **2**, **298**, and **363** displayed an inhibitory effect on α-glucosidase and were evaluated by p-nitro-phenyl-*α*-D-glucopyranoside as the substrate and acarbose as a positive control. Compound **2** showed a moderate inhibition, with IC_50_ values of 282 μM, surpassing the efficacy of the positive control acarbose (1.33 mM) [2]. Compound **298** displayed significant inhibition, with an IC_50_ value of 64.05 ± 1.59 μg/mL [100]. Compound **363** showed a moderate inhibition on *α*-glucosidase (IC_50_ = 7.94 μM). At the same time, the inhibition kinetics of compound **363** were studied via a noncompetitive inhibition mechanism, and the inhibition kinetics parameter (Ki) was 10.8 μM [120].

### 6.6. Cell Proliferation Activity

Compounds **100**, **477**, and **473** exhibited inhibitory effects on concanavalin A-induced T cell proliferation (IC_50_ = 13.6, 1.66, and 2.09 μM, respectively), as well as lipopolysaccharide-induced B cell proliferation (IC_50_ = 22.4, 1.37, and 3.31 μM, respectively), without exhibiting any obvious cytotoxicity to T cells and B cells [37,146]. Compounds **258** and **261** showed strong inhibitory activities against Con A-induced T lymphocyte proliferation, with IC_50_ values of 23.7 and 8.69 μM, respectively. The remarkable enhancement in activity can be attributed to the configurational inversion at C-5 in compound **261** [81].

Compound **515** has been confirmed to promote the proliferation and differentiation of umbilical cord-derived mesenchymal stem cells into keratinocyte-like cells at a concentration of 10 μM [158].

### 6.7. Other Activities

Compounds **34**, **43**, and **55** demonstrated the NGF-mediated promotion of neurite outgrowth on PC12 cells at a concentration of 10 μM [17]. Compound **509** showed a potent neuroprotective effect against a hydrogen peroxidation-induced reduction in cell viability in PC12 cells at a concentration of 1 μM [151,154]. Compound **58** displayed the most effective inhibitory effect on osteoclast differentiation, exhibiting IC_50_ values of 0.7 μM. It downregulated the expression levels of osteoclast-related genes and promoted the apoptosis of osteoclasts. Compounds **58**–**60** inhibited osteoclast formation, with IC_50_ ranging from 0.7 to 4.0 μM, thereby demonstrating their antiosteoporosis effects [23]. Compounds **95**–**97** showed potent biological activities against some marine organisms. Compound **97** was highly toxic to *A. salina,* with an LC_50_ value of 6.36 μM. Moreover, compound **96** displayed significant toxicity toward *C. marina* and *H. akashiwo* (LC_50_ = 0.81 and 2.88 μM, respectively), while compound **95** exhibited higher effectiveness against *Alexandrium* sp., with an LC_50_ value of 8.73 μM [36]. Compounds **143** and **152**–**154** selectively inhibited BChE, with IC_50_ values of 2.4, 7.9, 50.8, and 0.9 µM, respectively. Moreover, compounds **143** and **154** moderately inhibited AChE, with IC_50_ values of 329.8 μM and 342.9 μM, respectively [54]. Compounds **249**–**252** effectively inhibited Th17 differentiation, exhibiting IC_50_ values ranging from 2 to 18.07 μM. Compounds **250**–**251** were more effective than the positive control digoxin, which is a classical inhibitor of Th17 differentiation [80]. Compound **364** inhibited the acetyl transfer activity of *M. tuberculosis* GlmU, with an IC_50_ value of 41.85 μM, representing a novel therapeutic target for tuberculosis [120]. Compound **371** can significantly inhibit the formation of macrophage foam cells induced by oxidized low-density lipoprotein, suggesting its potential as a protective agent against atherosclerosis [134]. Compounds **454**–**456** displayed antifobrotic activities on TGF-*β*1-induced rat renal proximal tubular cells, effectively attenuating the excessive production of collagen I and *α*-SMA [141]. Compound **505** exhibited inhibitory activities against PTP1B and was a moderate time-dependent inactivator of PTP1B, with k_i_ value of 0.11 M^−1^ s^−1^ [6]. Compound **539** exhibited a mortality rate of 30% in *P. redivivus* and 28% in *C. elegans* within a 24 h period at a concentration of 400 mg/L, whereas the control group (5% acetone) only resulted in a mortality rate of 1.5% during the same time frame [161]. The anti-AD activity of compound **547** was comparatively weaker than memantine (*p* < 0.05), and it can be regarded as an anti-AD compound candidate [170].

## 7. Conclusions

This review summarized 557 compounds among C19, C18, C17, and C16 norditerpenes from 2010–2023. Obviously, C19 norditerpenes are the characteristic and main bioactive components of norditerpenes. Lamiaceae plants contain abundant norditerpenes, especially *Isodon* and *Salvia* genera. The Lamiaceae plant *Salvia miltiorrhiza* possesses abundant C19 norditerpenes, and the Cephalotaxaceae plant *Cephalotaxus fortunei* possesses abundant cephalotane-type norditerpenes.

Most norditerpenes exhibited anti-tumor, anti-inflammatory, anti-bacterial, and antioxidant properties, as well as inhibitory effects against HIV and *α*-glucosidase. Recent research suggests that norditerpenes may be a possibility for the future development of anti-tumor drugs. For example, euphorane C (**139**) inhibited the proliferation of K562 cells (IC_50_ = 3.59 µM) and provided the possibility for developing anti-leukemia drugs. Cephinoid H (**208**) isolated from *C. fortunei* showed the strongest cytotoxic activities, with IC_50_ values of 0.10, 0.13, and 0.14 µM against cell lines A549, Hela, and SGC-7901, respectively. It can be used as a candidate drug for treating various types of cancer. In Table 14, most norditerpenes have inhibitory effects on lung cancer, breast cancer, and cervical cancer cells. We should find appropriate targets to explore their mechanisms and focus on their in vivo activities in the future. With the rapid development of nanomaterials, we can combine effective norditerpenoids with them to improve their targeting and efficacy. Due to antibiotic resistance and side effects, it is meaningful to discover new antibiotics. Actinomadurol (**64**) has good antibacterial activity and provides the possibility for the discovery of antibiotics. At the same time, the structure–activity relationship was studied, which found that the hydroxyl group at C-7 affected antibacterial activity. CH_3_O-12 and the 3,20-epoxy moiety in 17-*nor*-pimarane diterpenes can function as activating groups, while the sugar moiety at C-2 might be an inactivated group. This is beneficial for the design and synthesis of new antibacterial drugs. Norditerpenes produce anti-inflammatory effects by influencing recognized markers of inflammatory processes such as IF, NO levels, nuclear factor kappa-B (NF-κB), and tumor necrosis factor-alpha (TGF-*α*). In the future, norditerpenes may be a useful and safe method for treating inflammatory diseases, such as rheumatoid arthritis, and can play a similar role as ibuprofen and dexamethasone.

Although current research has shown that these compounds exhibit various biological activities, most of the research mainly focuses on in vitro cell activity assays. It is necessary to investigate their in vivo activities. Further clinical trials are crucial to confirm the pharmacological effects of norditerpenes in order to fully illustrate their therapeutic effects on diseases. We hope that this review can promote research on norditerpenes.

## Figures and Tables

**Table 1:** Sources of 557 norditerpenes (families, genera, and species and the corresponding quantity of the compounds).

Family	Genus	Species	Quantity of Norditerpenes
Lamiaceae (143)	*Callicarpa*	*Callicarpa integerrima*	2
	*Teucrium*	*Teucrium viscidum*	3
	*Sideritis*	*Sideritis pullulans*	2
	*Leucas*	*Leucas zeylanica*	2
	*Isodon*	*Isodon rubescens*	4
		*Isodon eriocalyx*	1
		*Isodon pharicus*	1
		*Isodon phyllostachys*	2
		*Isodon rosthornii*	1
		*Isodon ternifolius*	1
	*Salvia*	*Salvia aethiopis*	1
		*Salvia deserta*	5
		*Salvia miltiorrhiza*	26
		*Salvia mirzayanii*	1
		*Salvia grandifolia*	4
		*Salvia leriifolia*	1
		*Salvia yunnanensis*	3
		*Salvia digitaloides*	17
		*Salvia rhytidea*	3
		*Salvia sahendica*	1
		*Salvia castanea*	4
		*Salvia przewalskii*	5
	*Perovskia*	*Perovskia abrotanoides*	17
		*Perovskia atriplicifolia*	6
Euphorbiaceae (75)	*Croton*	*Croton cajucara*	1
		*Croton haumanianus*	1
		*Croton yanhuii*	4
		*Croton euryphyllus*	7
		*Croton caudatus*	2
		*Croton cascarilloides*	2
		*Croton crassifolius*	4
	*Flueggea*	*Flueggea acicularis*	5
		*Flueggea virosa*	2
	*Baccaurea*	*Baccaurea ramiflora*	4
	*Jatropha*	*Jatropha podagrica*	2
	*Trigonostemon*	*Trigonostemon chinensis*	11
		*Trigonostemon flavidus*	4
		*Trigonostemon howii*	1
	*Malpighia*	*Malpighia emarginata*	3
	*Phyllanthus*	*Phyllanthus flexuosus*	1
	*Drypetes*	*Drypetes perreticulata*	4
	*Aleurites*	*Aleurites moluccanus*	2
	*Euphorbia*	*Euphorbia neriifolia*	6
		*Euphorbia royleana*	1
		*Euphorbia ebracteolata*	4
		*Euphorbia dracunculoides*	2
Cephalotaxaceae (66)	*Cephalotaxus*	*Cephalotaxus sinensis*	8
		*Cephalotaxus fortunei*	56
		*Cephalotaxus mannii*	2
Celastraceae (20)	*Euonymus* L.	*Euonymus verrucosus* var. *pauciflorus*	3
		*Euonymus grandiflflorus* Wall	1
	*Maytenus*	*Maytenus senegalensis*	1
	*Celastrus*	*Celastrus angulatus*	9
		*Celastrus orbiculatus*	4
	*Tripterygium*	*Tripterygium wilfordii*	2
Icacinaceae (16)	*Icacina*	*Icacina trichantha*	16
Orchidaceae (16)	*Flickingeria*	*Flickingeria fimbriata*	16
Podocarpaceae (14)	*Podocarpus*	*Podocarpus nagi*	9
		*Podocarpus macrophyllus*	5
Pinaceae (13)	*Abies*	*Abies forrestii*	2
	*Pinus*	*Pinus yunnanensis*	1
		*Pinus banksiana* Lamb	1
	*Pseudotsuga*	*Pseudotsuga sinensis*	9
Picrodendraceae (12)	*Austrobuxus*	*Austrobuxus carunculatus*	12
Taxaceae (11)	*Amentotaxus*	*Amentotaxus argotaenia*	11
Compositae (10)	*Eupatorium*	*Austroeupatorium inulifolium*	10
Chloranthaceae (9)	*Chloranthus*	*Chloranthus serratus*	2
		*Chloranthus sessilifolius*	7
Asteraceae (8)	*Grazielia*	*Grazielia gaudichaudeana*	3
	*Wedelia*	*Wedelia trilobata*	1
	*Stevia*	*Stevia rebaudiana*	3
	*Baccharis*	*Baccharis retusa*	1
Zingiberaceae (8)	*Amomum*	*Amomum maximum*	2
		*Amomum villosum*	1
	*Hedychium*	*Hedychium forrestii*	3
	*Elettaria*	*Elettaria cardamomum*	2
Salicaceae (7)	*Populus*	*Populus euphratica*	7
Araucariaceae (5)	*Agathis*	*Agathis macrophylla*	5
Leguminosae (5)	*Erythrophleum*	*Erythrophleum fordii*	2
		*Erythrophleum suaveolens*	1
	*Caesalpinia*	*Caesalpinia decapetala* var. *japonica*	1
		*Caesalpinia minax*	1
Menispermaceae (5)	*Tinospora*	*Tinospora capillipes*	5
Annonaceae (4)	*Annona*	*Annona squamosa* L.	2
	*Polyalthia*	*Polyalthia longifolia*	2
Dipterocarpaceae (4)	*Resina*	*Resina Commiphora*	4
Pallaviciniaceae (4)	*Pallavicinia*	*Pallavicinia ambigua*	4
Scrophulariaceae (4)	*Scrophularia*	*Scrophularia dentata*	4
Alangiaceae (2)	*Alangium*	*Alangium chinense* (Lour.) Harms	2
Botryosphaeriaceae (2)	*Diplodia*	*Diplodia olivarum*	2
Caprifoliaceae (2)	*Viburnum*	*Viburnum odoratissimum*	2
Ditrichaceae (2)	*Ceratodon*	*Ceratodon purpureus*	2
Malpighiaceae (2)	*Aspidopterys*	*Aspidopterys obcordata*	2
Paeoniaceae (2)	*Paeonia*	*Paeonia veitchii*	2
Rosaceae (2)	*Crataegus*	*Crataegus pinnatifida*	2
Thymelaeaceae (2)	*Aquilaria*	*Chinese eaglewood*	2
Apiaceae (1)	*Azorella*	*Azorella compacta*	1
Cupressaceae (1)	*Thuja*	*Thuja orientalis*	1
Ericaceae (1)	*Lyonia*	*Lyonia ovalifolia*	1
Pentaphylacaceae (1)	*Adinandra*	*Adinandra poilanei*	1
Ranunculaceae (1)	*Trollius*	*Trollius chinensis*	1
Eurotiaceae (35)	*Penicillium*	*Penicillium oxalicum*	1
		*Penicillium* sp. DT10	1
		*Penicillium thomii*	1
	*Aspergillus*	*Aspergillus taichungensis*	4
		*Aspergillus aculeatinus*	1
		*Aspergillus* sp. YXf3	1
		*Aspergillus wentii* EN-48	26
Discellaceae (14)	*Oidiodendron*	*Oidiodendron truncatum*	14
Dematiaceae (3)	*Alternaria*	*Alternaria brassicicola*	3
Thermomonosporaceae (2)	*Actinomadura*	*Actinomadura* sp. KC 191	2
Carboniaceae (1)	*Xylaria*	*Xylaria longipes*	1
Moniliaceae (1)	*Trichoderma*	*Trichoderma citrinoviride*	1
Cortinariaceae (1)	*Cortinarius*	*Cortinarius pyromyxa*	1
Alcyonidae (30)	*Sinularia*	*Sinularia scabra*	16
		*Sinularia maxima*	3
		*Sinularia nanolobata*	1
		*Sinularia siaesensis*	1
		*Sinularia hirta*	2
		*Sinularia densa*	7
Choiidae (9)	*Spongia*	*Spongia officinalis*	2
	*Dendrilla*	*Dendrilla antarctica*	2
	*Cacospongia*	*Cacospongia* sp.	5
Xeniidae (5)	*Cespitularia*	*Cespitularia taeniata*	2
		*Cespitularia* sp.	3
Chromodorididae (3)	*Goniobranchus*	*Goniobranchus Mollusks*	3
Gorgoniaceae (1)	*Junceella*	*Junceella fragilis*	1
Anthoptilidae (1)	*Anthoptilum*	*Anthoptilum grandiflflorum*	1
Loliginidae (1)	*Uroteuthis*	*Uroteuthis* (Photololigo) *duvaucelii*	1
Veneridae (1)	*Paphia*	*Paphia malabarica*	1
Amber (1)	*-*	*Dominican amber*	1

**Table 2:** Chemical constituents of labdane-type C19 norditerpenes.

No.	Name	Plant Source	Plant Organ	Ref.
1	penioxalicin	*P. oxalicum*	-	[2]
2	penitholabene	*P. thomii*	-	[3]
3	euonymupene C	*E. verrucosus*	twigs	[4]
4	euonymupene A	*E. verrucosus*	twigs	[4]
5	3*β*-hydroxy-15-*nor*-14-oxo-8(17),12-labdadien-14-al	*C. serratus*	whole plants	[5]
6	3*β*,6*β*-dihydroxy-15-*nor*-14-oxo-8(17),12-labdadien-14-al	*C. serratus*	whole plants	[5]
7	15-*nor*-14-oxolabda-8(17),12E-dien-19-oic acid	*A. macrophylla*	aerial parts	[6]
8	15-*nor*-14-oxolabda-8(17),13(16)-dien-19-oic acid	*T. orientalis*	leaves and stems	[7]
9	grazielabdane A	*G. gaudichaudeana*	aerial parts	[8]
10	grazielabdane B	*G. gaudichaudeana*	aerial parts	[8]
11	grazielabdane C	*G. gaudichaudeana*	aerial parts	[8]
12	austroeupatol	*A. inulifolium*	aerial parts	[9,10]
13	inulifolinone A	*A. inulifolium*	leaves	[9,10]
14	inulifolinone B	*A. inulifolium*	leaves	[9,10]
15	inulifolinone C	*A. inulifolium*	leaves	[9,10]
16	inulifolinone D	*A. inulifolium*	leaves	[9,10]
17	inulifolinone E	*A. inulifolium*	leaves	[9,10]
18	inulifolinone F	*A. inulifolium*	leaves	[9,10]
19	inulifolinone I	*A. inulifolium*	leaves	[9,10]
20	inulifolinone G	*A. inulifolium*	leaves	[9,10]
21	inulifolinone H	*A. inulifolium*	leaves	[9,10]
22	amomaxin B	*A. maximum*	roots	[11]
23	amomaxin A	*A. maximum*	roots	[11]
24	hedychin E	*H. forrestii*	rhizomes	[12]
25	pallambin A	*P. ambigua*	-	[13]
26	pallambin B	*P. ambigua*	-	[13]
27	pallambin C	*P. ambigua*	-	[13]
28	pallambin D	*P. ambigua*	-	[13]

**Table 3:** Chemical constituents of clerodane-type C19 norditerpenes.

No.	Name	Plant Source	Plant Organ	Ref.
29	15-*nor*-cleroda-3,12-diene	*D. amber*	-	[14]
30	callinteger A	*C. integerrima*	twigs and leaves	[15]
31	callinteger B	*C. integerrima*	twigs and leaves	[15]
32	*trans*-dehydrocrotonin	*C. cajucara*	tree	[16]
33	croyanoid *B*	*C. yanhuii*	twigs and leaves	[17]
34	crotoeurin B	*C. euryphyllus*	twigs and leaves	[18]
35	crocleropene B	*C. caudatus*	twigs and leaves	[19]
36	crocleropene A	*C. caudatus*	twigs and leaves	[19]
37	croyanoid C	*C. yanhuii*	twigs and leaves	[17]
38	tinocapillin A	*T. capillipes*	rhizomes	[20]
39	tinocallone A	*T. capillipes*	rhizomes	[20]
40	tinocallone C	*T. capillipes*	rhizomes	[20]
41	tinocapillin B	*T. capillipes*	rhizomes	[20]
42	tinocapillin C	*T. capillipes*	rhizomes	[20]
43	crotoeurin C	*C. euryphyllus*	twigs and leaves	[18]
44	6-*epi*-crotoeurin C	*C. crassifolius*	roots	[21]
45	teucvidin	*C. euryphyllus*	twigs and leaves	[18]
46	isoteucvin	*C. euryphyllus*	twigs and leaves	[18]
47	teucvin	*C. euryphyllus*	twigs and leaves	[17,18]
48	isocrotocaudin	*C. euryphyllus*	twigs and leaves	[17,18]
49	crotocaudin	*C. crassifolius*	roots	[21]
50	croyanoid D	*C. yanhuii*	twigs and leaves	[17]
51	teucvisin C	*T. viscidum*	whole plants	[22]
52	teucvisin D	*T. viscidum*	whole plants	[22]
53	teucvisin E	*T. viscidum*	whole plants	[22]
54	croyanoid A	*C. yanhuii*	twigs and leaves	[17]
55	crotoeurin A	*C. euryphyllus*	twigs and leaves	[17,18]

**Table 4:** Chemical constituents of pimarane-type C19 norditerpenes.

No.	Name	Plant Source	Plant Organ	Ref.
56	fluacinoid D	*F. acicularis*	aerial parts	[23]
57	fluacinoid E	*F. acicularis*	aerial parts	[23]
58	fluacinoid F	*F. acicularis*	aerial parts	[23]
59	fluacinoid G	*F. acicularis*	aerial parts	[23]
60	fluacinoid H	*F. acicularis*	aerial parts	[23]
61	aquilariaene F	*C. eaglewood*	-	[24]
62	aquilariaene H	*C. eaglewood*	-	[24]
63	jbir-65	*Actinomadura* sp. KC 191	-	[25]
64	actinomadurol	*Actinomadura* sp. KC 191	-	[25]
65	icatrichanone	*I. trichantha*	tubers	[26]
66	14-hydroxyicatrichanone	*I. trichantha*	tubers	[26]
67	3-*O*-methylicatrichanone	*I. trichantha*	tubers	[26]
68	3-*O*-methyl-14-hydroxyicatrichanone	*I. trichantha*	tubers	[26]
69	7*α*-hydroxyicacenone	*I. trichantha*	tubers	[27]
70	icacenone	*I. trichantha*	tubers	[27]
71	12-hydroxy-icacinlactone	*I. trichantha*	tubers	[27]
72	icacinlactone H	*I. trichantha*	tubers	[27]
73	icacinlactone A	*I. trichantha*	tubers	[28]
74	icacinlactone B	*I. trichantha*	tubers	[27,28]
75	icacinlactone E	*I. trichantha*	tubers	[28]
76	icacinlactone F	*I. trichantha*	tubers	[28]
77	icacinlactone G	*I. trichantha*	tubers	[28]
78	icacinlactone C	*I. trichantha*	tubers	[28]
79	icacinlactone D	*I. trichantha*	tubers	[27,28]
80	trichanthol B	*I. trichantha*	tubers	[27]
81	8*β*-hydroxy-18-*nor*-4(5),15-isopimaradien-3-one	*E. grandiflflorus* Wall.	branches and leaves	[29]
82	18(4→14), 19(4→8)-bis-abeo-*nor*-isopimarane-1,5-diene-3-yl-3*β*-methoxy propyl pentanoate	*P. malabarica*	edible portion	[30]
83	smardaesidin F	*C. purpureus*	-	[31]
84	smardaesidin G	*C. purpureus*	-	[31]
85	sphaeropsidin G	*D. olivarum*	-	[32]
86	taichunin D	*A. taichungensis*	-	[33]
87	aspewentin D	*A. wentii* EN-48	-	[34]
88	aspewentin E	*A. wentii* EN-48	-	[34]
89	aspewentin F	*A. wentii* EN-48	-	[34]
90	aspewentin I	*A. wentii* EN-48	-	[35]
91	aspewentin J	*A. wentii* EN-48	-	[35]
92	aspewentin K	*A. wentii* EN-48	-	[35]
93	aspewentin M	*A. wentii* EN-48	-	[35]
94	aspewentin L	*A. wentii* EN-48	-	[35]
95	aspewentin C	*A. wentii* EN-48	-	[36]
96	aspewentin A	*A. wentii* EN-48	-	[36]
97	aspewentin B	*A. wentii* EN-48	-	[36]
98	aspewentin G	*A. wentii* EN-48	-	[34]
99	aspewentin H	*A. wentii* EN-48	-	[34]
100	xylarilongipin A	*A. wentii* EN-48	-	[37]
101	asperether A	*A. wentii* EN-48	-	[38]
102	asperether B	*A. wentii* EN-48	-	[38]
103	asperether C	*A. wentii* EN-48	-	[38]
104	asperether D	*A. wentii* EN-48	-	[38]
105	asperether E	*A. wentii* EN-48	-	[38]
106	(2*S*,3*S*,5*S*,9*S*,10*S*,13*S*)-2,3-dihydroxy-16-*nor*-*ent*-pimar-8(14)-en-15-oic acid	*F. fimbriata*	aerial parts	[39]
107	(3*R*,5*S*,9*S*,10*S*,13*S*)-2-hydroxy-16-*nor*-*ent*-pimar-8(14)-en-15-oic acid	*F. fimbriata*	aerial parts	[39]
108	(2*S*,3*R*,5*S*,9*S*,10*S*,13*S*)-2-acetoxy-3-hydroxy-16-*nor*-*ent*-pimar-8(14)-en-15-oic acid	*F. fimbriata*	aerial parts	[39]
109	norflickinflimiod A	*F. fimbriata*	aerial parts	[39,40]
110	(2*S*,3*R*,5*S*,9*S*,10*S*,13*S*)-2-*O*-*E*-cinnamoyl-3-hydroxy-16-*nor*-*ent*-pimar-8(14)-en-15-oic acid	*F. fimbriata*	aerial parts	[39]
111	3*α*,14*β*-diacetoxy-16-*nor*-*ent*-pimar-15*α*,8-olide	*F. fimbriata*	aerial parts	[39]
112	norflickinflimiod B	*F. fimbriata*	aerial parts	[39,40]
113	norflickinflimiod C	*F. fimbriata*	aerial parts	[40]
114	norflickinflimoside	*F. fimbriata*	aerial parts	[40]
115	norflickinflimiod D	*F. fimbriata*	aerial parts	[40]
116	eupneria K	*E. neriifolia*	stem bark	[41]
117	eupneria L	*E. neriifolia*	stem bark	[41]
118	eupneria J	*E. neriifolia*	stem bark	[41]
119	eupneria M	*E. neriifolia*	stem bark	[41]
120	(3*R*,4*R*,5*S*,9*R*,10*S*,12*S*,13*S*)-*ent*-18-*nor*-8(14),15-isopimaradiene-3*β*,12*β*,4*α*-triol	*E. neriifolia*	stems	[42]

**Table 5:** Chemical constituents of abietane-type C19 norditerpenes.

No.	Name	Plant Source	Plant Organ	Ref.
121	dichroanone	*S. deserta*	roots	[43]
122	salviadesertin A	*S. deserta*	roots	[43]
123	salviadesertin B	*S. deserta*	roots	[43]
124	salviadesertin E	*S. deserta*	roots	[43]
125	salviadesertin F	*S. deserta*	roots	[43]
126	dehydromiltirone	*S. miltiorrhiza*	cell	[44]
127	grandifolia D	*S. grandifolia*	roots	[45]
128	2-isopropyl-8,8-dimethyl-7,8-dihydroph-enanthrene-1,4,5(6*H*)-trione	*S. leriifolia*	whole plants	[46]
129	deoxyneocryptotanshinone	*S. rhytidea*	roots	[47]
130	(1*R*,15*R*)-1*β*-hydroxyneocryptotanshinone.	*P. abrotanoides*	roots	[48]
131	19-*nor*-abieta-4,6,8,11,13-tetraen-3-one	*P. euphratica*	resins	[49]
132	4,4*α*,9,10-tetrahydro-1,4*α*-dimethyl-7-isopropyl-2(3*H*)-phenanthrone	*P. euphratica*	resins	[49]
133	19-*nor*-abieta-4(18),8,11,13-tetraen-7-one	*P. euphratica*	resins	[49]
134	castanol B	*S. castanea*	whole plants	[50]
135	triptobenzene S	*T. wilfordii*	roots	[51]
136	1-deoxo aurocadiol	*S. rhytidea*	roots	[47]
137	arucadiol	*S. rhytidea*	roots	[47]
138	1-deoxy-1,2-dien-3-oxoarucadiol.	*P. abrotanoides*	roots	[48]
139	euphorane C	*E. dracunculoides*	powder	[52]
140	nagiol A	*P. nagi*	leaves	[53]
141	18-norabieta-8,11,13-4-ol	*P. euphratica*	resins	[49]
142	grandifolia A	*S. grandifolia*	roots	[45]
143	isograndifoliol	*P. atriplicifolia*	roots	[45,54]
144	grandifolia B	*S. grandifolia*	roots	[45]
145	przewalskin Y-1	*P. abrotanoides*	roots	[48]
146	miltipolone	*S. grandifolia*	roots	[45]
147	dehydrodanshenol A	*S. przewalskii*	roots	[55]
148	cryptotanshinone	*S. miltiorrhiza*	seeds	[44,56]
149	tanshinone IIA	*S. miltiorrhiza*	seeds	[56]
150	methyltanshinoate	*S. castanea*	whole plants	[50]
151	1-oxocryptotanshinone	*P. atriplicifolia*	roots	[48]
152	(1*R*,15*R*)-1-acetoxycryptotanshinone	*P. atriplicifolia*	roots	[54]
153	(1*R*)-1-acetoxytanshinone IIA	*P. atriplicifolia*	roots	[54]
154	(15*R*)-1-oxoaegyptinone A	*P. atriplicifolia*	roots	[54]
155	rubesanolide F	*I. rubescens*	leaves	[57]
156	rubesanolide G	*I. rubescens*	leaves	[57]
157	castanol A	*S. castanea*	whole plants	[50]
158	abieseconordine A	*A. forrestii*	twigs	[58]
159	abieseconordine B	*A. forrestii*	twigs	[58]
160	5-(6-isopropyl-2-methylnaphthalen-1-yl)pentan-2-one	*P. euphratica*	resins	[49]
161	deacetylsalvianonol	*S. przewalskii*	roots	[55]
162	sessilifol O	*C. sessilifolius*	whole plants	[59]
163	salvialba acid	*S. miltiorrhiza*	roots	[60]
164	salprzelactone	*S. przewalskii*	roots	[55]

**Table 6:** Chemical constituents of kaurane-type C19 norditerpenes.

No.	Name	Plant Source	Plant Organ	Ref.
165	laxiflorolide M	*I. eriocalyx*	leaves	[61]
166	ternifolide B	*I. ternifolius*	-	[62]
167	*ent*-16-oxo-17-*nor*-kauran-19-oic	*B. retusa*	aerial parts	[63]
168	sideritone A	*S. pullulans*	aerial parts and roots	[64]
169	sideritone B	*S. pullulans*	aerial parts and roots	[64]
170	amentotaxin L	*A. argotaenia*	twigs and leaves	[65]
171	18-*nor*-*ent*-kaur-4(19)-en-17-oic acid	*M. senegalensis*	root bark	[66]
172	16*α*,17,19-trihydroxy-18-*nor*-*ent*-kauran-4*β*-ol	*W. trilobata*	whole plants	[67]
173	amentotaxin M	*A. argotaenia*	twigs and leaves	[65]
174	amentotaxin C	*A. argotaenia*	twigs and leaves	[65]
175	amentotaxin D	*A. argotaenia*	twigs and leaves	[65]
176	amentotaxin E	*A. argotaenia*	twigs and leaves	[65]
177	amentotaxin F	*A. argotaenia*	twigs and leaves	[65]
178	amentotaxin G	*A. argotaenia*	twigs and leaves	[65]
179	amentotaxin H	*A. argotaenia*	twigs and leaves	[65]
180	amentotaxin I	*A. argotaenia*	twigs and leaves	[65]
181	amentotaxin J	*A. argotaenia*	twigs and leaves	[65]
182	amentotaxin K	*A. argotaenia*	twigs and leaves	[65]
183	19-*nor*-16,17-dihydroxy-*ent*-kaur-4(18)-ene	*C. haumanianus*	leaves and stem bark	[68]
184	wilkaunoid D	*T. wilfordii*	stems	[69]
185	(4*α*)-19-nor-*ent*-kaurane-4,16,17-triol	*A. squamosa* L.	stem bark	[70]
186	(4*α*,16*α*)-17-(acetyloxy)-19-*nor*-*ent*-kaurane-4,16-diol	*A. squamosa* L.	stem bark	[70]
187	phyllostachysin N	*I. phyllostachys*	aerial parts	[71]
188	phyllostachysin O	*I. phyllostachys*	aerial parts	[71]
189	isorosthin A	*I. rosthornii*	aerial parts	[72]

**Table 7:** Chemical constituents of cephalotane-type C19 norditerpenes.

No.	Name	Plant Source	Plant Organ	Ref.
190	cephalotanin A	*C. sinensis*	leaves	[73]
191	cephalotanin B	*C. sinensis*	leaves	[73]
192	cephalotanin C	*C. sinensis*	leaves	[73]
193	ceforalide F	*C. fortunei*	seeds	[74]
194	ceforalide G	*C. fortunei*	seeds	[74]
195	ceforalide H	*C. fortunei*	seeds	[74]
196	cephalotanin D	*C. sinensis*	leaves	[73]
197	cephanolide D	*C. sinensis*	twigs and leaves	[75]
198	ceforalide A	*C. fortunei*	seeds	[74]
199	ceforalide B	*C. fortunei*	seeds	[74]
200	ceforalide C	*C. fortunei*	seeds	[74]
201	ceforalide D	*C. fortunei*	seeds	[74]
202	ceforalide E	*C. fortunei*	seeds	[74]
203	fortalpinoid P	*C. fortunei*	seeds	[76]
204	fortalpinoid Q	*C. fortunei*	seeds	[76]
205	20-oxohainanolidol	*C. fortunei*	twigs and leaves	[77]
206	20*α*-hydroxyhainanolidol	*C. fortunei*	twigs and leaves	[77]
207	10-hydroxyhainanolidol	*C. fortunei*	twigs and leaves	[77]
208	cephinoid H	*C. fortunei*	twigs and leaves	[78]
209	cephinoid I	*C. fortunei*	twigs and leaves	[78]
210	cephinoid J	*C. fortunei*	twigs and leaves	[78]
211	cephinoid K	*C. fortunei*	twigs and leaves	[78]
212	cephinoid L	*C. fortunei*	twigs and leaves	[78]
213	cephinoid M	*C. fortunei*	twigs and leaves	[78]
214	hainanolidol	*C. fortunei*	twigs and leaves	[78]
215	fortunolide A	*C. fortunei*	twigs and leaves	[78]
216	fortalpinoid A	*C. fortunei*	seeds	[76]
217	fortalpinoid B	*C. fortunei*	seeds	[76]
218	fortalpinoid C	*C. fortunei*	seeds	[76]
219	fortalpinoid D	*C. fortunei*	seeds	[76]
220	fortalpinoid E	*C. fortunei*	seeds	[76]
221	fortalpinoid F	*C. fortunei*	seeds	[76]
222	3-deoxyfortalpinoid F	*C. fortunei*	seeds	[76]
223	10-hydroxyharringtonolide	*C. mannii*	twigs and leaves	[78,79]
224	cephinoid F	*C. fortunei*	twigs and leaves	[78]
225	cephinoid G	*C. fortunei*	twigs and leaves	[78]
226	harringtonolide	*C. fortunei*	twigs and leaves	[78]
227	fortunolide B	*C. fortunei*	twigs and leaves	[78]
228	fortalpinoid J	*C. fortunei*	seeds	[76]
229	6-en-harringtonolide	*C. mannii*	twigs and leaves	[78,79]
230	fortalpinoid H	*C. fortunei*	seeds	[76]
231	fortalpinoid I	*C. fortunei*	seeds	[76]
232	cephanolide J	*C. fortunei*	seeds	[76]
233	fortalpinoid G	*C. fortunei*	seeds	[76]
234	cephinoid N	*C. fortunei*	twigs and leaves	[78]
235	cephinoid O	*C. fortunei*	twigs and leaves	[78]
236	fortalpinoid M	*C. fortunei*	seeds	[76]
237	fortalpinoid N	*C. fortunei*	seeds	[76]
238	fortalpinoid O	*C. fortunei*	seeds	[76]
239	cephafortoid A	*C. fortunei*	twigs and leaves	[77]
240	14-*epi*-cephafortoid A	*C. fortunei*	twigs and leaves	[77]
241	cephinoid P	*C. fortunei*	twigs and leaves	[78]
242	cephinoid Q	*C. fortunei*	twigs and leaves	[78]
243	cephinoid R	*C. fortunei*	twigs and leaves	[78]
244	cephinoid S	*C. fortunei*	twigs and leaves	[78]
245	gongshanolide	*C. fortunei*	twigs and leaves	[78]
246	fortalpinoid K	*C. fortunei*	seeds	[76]
247	fortalpinoid L	*C. fortunei*	seeds	[76]
248	ceforalide I	*C. fortunei*	seeds	[74]
249	cephalodione A	*C. fortunei*	seeds	[80]
250	cephalodione B	*C. fortunei*	seeds	[80]
251	cephalodione C	*C. fortunei*	seeds	[80]
252	cephalodione D	*C. fortunei*	seeds	[80]

**Table 8:** Chemical constituents of cembranoid-type C19 norditerpenes.

No.	Name	Plant Source	Plant Organ	Ref.
253	xiguscabrolide H	*S. scabra*	-	[81]
254	10-*epi*-gyrosanolide E	*S. scabra*	-	[81]
255	5-*epi*-sinuleptolide	*S. scabra*	-	[81]
256	norcembrene 5	*S. scabra*	-	[81]
257	scabrolide D	*S. scabra*	-	[81]
258	scabrolide G	*S. scabra*	-	[81]
259	sinularcasbane O	*S. scabra*	-	[81]
260	gyrosanolide F	*S. scabra*	-	[81]
261	sinuleptolide	*S. scabra*	-	[81]
262	5-*epi*-norcembrene	*S. maxima*	-	[82]
263	13-*epi*-scabrolide C	*S. maxima*	-	[82]
264	sinudenoid A	*S. densa*	-	[83]
265	sinudenoid B	*S. densa*	-	[83]
266	sinudenoid C	*S. densa*	-	[83]
267	sinudenoid D	*S. densa*	-	[83]
268	ineleganolide	*S. maxima*	-	[81,82,84,85]
269	fragilolide A	*J. fragilis*	-	[86]
270	sinuscalide C	*S. scabra*	-	[87]
271	sinuscalide D	*S. scabra*	-	[87]
272	sinuscalide B	*S. scabra*	-	[87]
273	scabrolide B	*S. densa*	-	[83]
274	scabrolide A	*S. densa*	-	[83]
275	sinudenoid E	*S. densa*	-	[83]
276	sinuscalide A	*S. scabra*	-	[87]

**Table 9:** Chemical constituents of other compounds of C19 norditerpenes.

No.	Name	Plant Source	Plant Organ	Ref.
277	japodagricanone A	*J. podagrica*	twigs and leaves	[88]
278	japodagricanone B	*J. podagrica*	twigs and leaves	[88]
279	6-((*E*)-12-(furan-13-yl)-10-methylpent-10-en-9-yl)-6,7,8,8atetrahydro-3*H*-isochromen-1-(5*H*)-one	*U.* (Photololigo) *duvaucelii*	-	[89]
280	erythro-norcassanoid A	*E. fordii*	roots	[90]
281	erythro-norcassanoid B	*E. fordii*	roots	[90]
282	caesalpinone	*C. decapetala* var. *japonica*	roots	[91]
283	aspidoptoid A	*A. obcordata*	vine	[92]
284	aspidoptoid B	*A. obcordata*	vine	[92]
285	phyllanflexoid C	*P. flexuosus*	roots	[93]
286	olicleistanone	*D. olivarum*	-	[32]
287	alterbrassicicene B	*A. brassicicola*	-	[94]
288	1*β*-hydroxy-brassicicene Q	*A. brassicicola*	-	[94]
289	3-ketobrassicicene W	*A. brassicicola*	-	[94]
290	aculeaterpene A	*A. aculeatinus*	-	[95]
291	dongtingnoid C	*P.* sp. DT10	-	[96]
292	crotocascarin *α*	*C. cascarilloides*	stems	[97]
293	crotocascarin *β*	*C. cascarilloides*	stems	[97]
294	(+)-paeoveitol	*P. veitchii*	roots	[98]
295	(−)-paeoveitol	*P. veitchii*	roots	[98]
296	eurifoloid M	*E. neriifolia*	twigs and leaves	[99]
297	eurifoloid P	*E. neriifolia*	twigs and leaves	[99]
298	adipoiloside	*A. poilanei*	leaves	[100]
299	citrinovirin	*T. citrinoviride*	-	[101]
300	16-deacetoxy-9,11-dihydrogracilin A	*G. Mollusks*	-	[102]
301	15,16-deacetoxy-15-hydroxy-9,11-dihydrogracilin A	*G. Mollusks*	-	[102]
302	verrielactone	*G. Mollusks*	-	[102]
303	3-*nor*-spongiolide A	*S. officinalis*	-	[103]
304	3-*nor*-spongiolide B	*S. officinalis*	-	[103]
305	scrodentoid F	*S. dentata*	whole plants	[104]
306	scrodentoid G	*S. dentata*	whole plants	[104]
307	scrodentoid H	*S. dentata*	whole plants	[104,105]
308	scrodentoid I	*S. dentata*	whole plants	[104,105]
309	normulin-11-en-13-oxo-20-oic acid	*A. compacta*	aerial part	[106]
310	pyromyxone D	*C. pyromyxa*	fruiting bodies	[107]
311	adenica	*L. zeylanica*	whole plants	[108]
312	6*β*-acetoxy-9*α*,13-epoxy-16-norlabd-13*E*-en-15-al	*L. zeylanica*	whole plants	[108]
313	scabrolide A	*S. scabra*	-	[81]
314	yonarolide	*S. scabra*	-	[81]
315	12-hydroxy-scabrolide A	*S. scabra*	-	[81]
316	sinusiaetone A	*S. siaesensis*	-	[109]
317	cespitaenin A	*C. taeniata*	-	[110]
318	cespitaenin B	*C. taeniata*	-	[110]
319	cespitulin Q	*Cespitularia* sp.	-	[111]
320	cespitulin R	*Cespitularia* sp.	-	[111]
321	cespitulin P	*Cespitularia* sp.	-	[111]
322	norhawthornoid A	*C. pinnatifida*	leaves	[112]
323	norhawthornoid B	*C. pinnatifida*	leaves	[112]
324	ebractenoid A	*E. ebracteolata*	roots	[113]
325	ebractenoid B	*E. ebracteolata*	roots	[113]
326	(2*S*,7*S*,11*S*)-(8*E*,12*Z*)-2, 10-dihydroxy-pellialactone	*A. chinense*	roots	[114]
327	(2*S*,4*S*,7*S*,11*S*)-(8*E*,12*Z*)-2,4,10-trihydroxy-pellialactone	*A. chinense*	roots	[114]
328	miltiolactone A	*S. miltiorrhiza*	roots	[115]
329	miltiolactone B	*S. miltiorrhiza*	roots	[115]
330	taichunin A	*A. taichungensis*	-	[33]
331	taichunin B	*A. taichungensis*	-	[33]
332	taichunin C	*A. taichungensis*	-	[33]
333	PR 1388	*O. truncatum*	-	[116]
334	oidiolactone D	*O. truncatum*	-	[116]
335	oidiolactone C	*O. truncatum*	-	[116]
336	oidiolactone G	*O. truncatum*	-	[116]
337	*epi*-oidiolactone G	*O. truncatum*	-	[116]
338	oidiolactone H	*O. truncatum*	-	[116]
339	oidiolactone I	*O. truncatum*	-	[116]
340	oidiodendronic acid	*O. truncatum*	-	[116]
341	oidiolactone E	*O. truncatum*	-	[116]
342	LL-Z1271*α*	*O. truncatum*	-	[116]
343	oidiolactone J	*O. truncatum*	-	[116]
344	oidiolactone K	*O. truncatum*	-	[116]
345	oidiolactone L	*O. truncatum*	-	[116]
346	LL-Z1271*β*	*O. truncatum*	-	[116]
347	16-*epi*-pretoxin	*A. carunculatus*	fruits	[117]
348	austrobuxusin F	*A. carunculatus*	fruits	[117]
349	austrobuxusin G	*A. carunculatus*	fruits	[117]
350	austrobuxusin H	*A. carunculatus*	fruits	[117]
351	16-*epi*-austrobuxusin H	*A. carunculatus*	fruits	[117]
352	16-*epi*-austrobuxusin G	*A. carunculatus*	fruits	[117]
353	austrobuxusin I	*A. carunculatus*	fruits	[117]
354	austrobuxusin J	*A. carunculatus*	fruits	[117]
355	16-*epi*-austrobuxusin B	*A. carunculatus*	fruits	[117]
356	austrobuxusin K	*A. carunculatus*	fruits	[117]
357	austrobuxusin L	*A. carunculatus*	fruits	[117]
358	austrobuxusin M	*A. carunculatus*	fruits	[117]
359	methyl-13-acetyl-podocarpa-8,11,13-trien-18-oate	*P. euphratica*	resins	[49]
360	sinuhirtone B	*S. hirta*	-	[118]
361	9,11-dihydrogracilin A	*D. antarctica*	-	[119]
362	9,11-dihydrogracillinone A	*D. antarctica*	-	[119]
363	eupractenoid A	*E. ebracteolata*	roots	[120]
364	eupractenoid B	*E. ebracteolata*	roots	[120]

**Table 10:** Chemical constituents of C18 norditerpenes.

No.	Name	Plant Source	Plant Organ	Ref.
365	salyunnanin F	*S. yunnanensis*	roots	[121]
366	16,17-dinorpisferal A	*S. digitaloides*	roots	[122]
367	militibetin A	*S. miltiorrhiza*	dry roots	[48]
368	(5*S*,8*S*,10*R*)-militibetinA	*P. abrotanoides*	roots	[48]
369	normiltioane	*S. miltiorrhiza*	cell cultures	[44]
370	yunnannin A	*S. miltiorrhiza*	dry roots	[123]
371	(5*S*,6*S*,7*R*,10*R*)-16,17-bis-*nor*-7α-hydroxy-18,6-epoxyferruginol	*P. abrotanoides*	roots	[48]
372	(5*S*,6*S*,7*S*)-16,17-bis-*nor*-7α-hydroxyferruginol-18,6-olide	*P. abrotanoides*	roots	[48]
373	przewalskin	*S. digitaloides*	roots	[122]
374	(5*S*,6*S*,7*R*,10*R*)-16,17-bis-*nor*-6*β*-hydroxy-18,7-epoxyferruginol	*P. abrotanoides*	roots	[48]
375	(5*R*,7*S*,10*R*)-3-oxoprzewalskin	*P. abrotanoides*	roots	[48]
376	grandifolia C	*P. abrotanoides*	roots	[48]
377	2-isopropyl-8-methylphenan-threne-3,4-dione	*S. miltiorrhiza*	cell cultures	[44]
378	(5*S*,10*R*)-16,17-bis-*nor*-pisiferanol	*P. abrotanoides*	roots	[48]
379	5*S*-1,2-dihydroheudelotinol	*T. chinensis*	stem bark and wood	[124]
380	deoxofaveline	*P. abrotanoides*	roots	[48]
381	trigonostemon A	*T. chinensis*	stem bark and wood	[124]
382	trigonostemon B	*T. chinensis*	stem bark and wood	[124]
383	5*S*-heudelotinone	*T. chinensis*	stem bark and wood	[124]
384	trigonostemon C	*T. chinensis*	stem bark and wood	[124]
385	trigonostemon D	*T. chinensis*	stem bark and wood	[124]
386	heudelotinone	*T. howii*	stems and leaves	[124]
387	norperovskatone	*P. atriplicifolia*	flowers	[125]
388	salviolone	*P. abrotanoides*	roots	[48]
389	*epi*-castanolide	*P. abrotanoides*	roots	[48]
390	cryptoacetalide	*S. miltiorrhiza*	cell cultures	[44]
391	epicryptoacetalide	*S. miltiorrhiza*	cell cultures	[44]
392	*epi*-danshenspiroketallactone A	*S. miltiorrhiza*	cell cultures	[44]
393	tanshinone I	*S. digitaloides*	cell cultures	[56,122]
394	dihydrotanshinone I	*S. miltiorrhiza*	cell cultures	[56]
395	dihydrotanshinone	*S. digitaloides*	roots	[122]
396	dihydroisotanshinone I	*S. miltiorrhiza*	cell cultures	[44]
397	tanshinketolactone	*S. miltiorrhiza*	cell cultures	[44]
398	trigonostemon G	*T. chinensis*	stem bark	[126]
399	trigonostemon H	*T. chinensis*	stem bark	[126]
400	flueggenoid E	*S. miltiorrhiza*	roots and rhizomes	[127]
401	12-hydroxy-20(10→5)-abeo-4,5-*seco*-podocarpa-5(10),6,8,11,13-pentaen-3-one	*S. miltiorrhiza*	roots and rhizomes	[127]
402	3*β*,12-dihydroxy-13-methylpodocarpa-6,8,11,13-tetraene	*S. digitaloides*	roots	[128]
403	3α-hydroxy-12-methoxy-13-methyl-*ent*-podocar-6,8,11,13-tetraene	*S. digitaloides*	roots	[128]
404	12-hydroxy-13-methyl-*ent*-podocarp-6,8,11,13-tetraen-3-one	*S. digitaloides*	roots	[128]
405	12-methoxy-13-methyl-*ent*-podocarp-6,8,11,13-tetraen-3-one	*S. digitaloides*	roots	[128]
406	jatromulone A	*T. chinensis*	stem bark and wood	[129]
407	gossweilone	*T. chinensis*	stem bark and wood	[129]
408	flueggenoid C	*S. miltiorrhiza*	roots and rhizomes	[127]
409	3*β*,12-dihydroxy-13-methylpodocarpane-8,10,13-triene	*T. chinensis*	stem bark and wood	[129]
410	3*β*,12-dihydroxy-13-methylpodocarpa-8,11,13-triene	*S. digitaloides*	roots	[128]
411	3*α*-hydroxy-12-methoxy-13-methyl-ent-podocarpa-6,8,11,13-tetraene	*S. miltiorrhiza*	roots and rhizomes	[127]
412	3*α*,12-dihydroxy-13-methyl-ent-podocarpa-6,8,11,13-tetraene	*S. miltiorrhiza*	roots and rhizomes	[127]
413	6*β*,12-dihydroxy-13-methyl-ent-podocarp-8,11,13-trien-3-one	*S. digitaloides*	roots	[128]
414	rel-(5*β*,8*α*,10*α*)-8-hydroxy-13-methylpodocarpa-9(11),13-diene-3,12-dione	*A. moluccanus*	twigs	[130]
415	3*α*-hydroxy-13-hydroxymethyl-12-methoxy-ent-podocarp-6,8,11,13-tetraene	*S. digitaloides*	roots	[128]
416	3*β*-hydroxy-13-hydroxymethyl-12-methoxy-ent-podocarp-6,8,11,13-tetraene	*S. digitaloides*	roots	[128]
417	10*α*,12-dihydroxy-13-methyl-9(10→20)-abeo-*ent*-podocarpa-6,8,11,13-tetraen-3-one	*S. miltiorrhiza*	roots and rhizomes	[127]
418	flueggenoid A	*S. miltiorrhiza*	roots and rhizomes	[127]
419	flueggenoid.B	*S. miltiorrhiza*	roots and rhizomes	[127]
420	flueggenoid D	*S. miltiorrhiza*	roots and rhizomes	[127]
421	6,12-dihydroxy-13-methyl-7-oxo-*ent*-podocarpa-5,8,11,13-tetraeno-20,3*α*-lactone	*S. miltiorrhiza*	roots and rhizomes	[127]
422	3*α*,20-epoxy-3β-hydroxy-12-methoxy-13-methyl-*ent*-podocarp-8,11,13-triene	*S. digitaloides*	roots	[128]
423	7*α*,20-epoxy-3*α*-hydroxy-12-methoxy-13-methyl-*ent*-podocarp-8,11,13-triene	*S. digitaloides*	roots	[128]
**424**	3*β*-hydroxymakilactone A	*P. macrophyllus*	roots	[131]
**425**	2*β*-hydroxymakilactone A	*P. macrophyllus*	roots	[131]
426	inumakilactone A	*P. macrophyllus*	roots	[131]
427	makilactone M	*P. macrophyllus*	roots	[131]
428	inumakilactone B	*P. macrophyllus*	roots	[131]
429	(4*S*,5*R*,9*S*,10*R*)-methyl-19-hydroxy-15,16-dinorlabda-8(17),11*E*-dien-13-oxo-18-oate	*A. macrophylla*	-	[6]
430	pseudosinin C	*P. sinensis*	needles and twigs	[132]
431	pseudosinin B	*P. sinensis*	needles and twigs	[132]
432	8*α*-hydroxy-11(*E*)-en-13-oxo-14,15-dinorlabdan-18-oic	*S. mirzayanii*	-	[133]
433	austroinulin	*S. rebaudiana*	aerial parts	[134]
434	sterebin A	*S. rebaudiana*	aerial parts	[134]
435	sterebin D	*S. rebaudiana*	aerial parts	[134]
436	hedychin A	*H. forrestii*	rhizomes	[12]
437	hedychin F	*H. forrestii*	rhizomes	[12]
438	lyonivaloside I	*L. ovalifolia*	twigs and leaves	[135]
439	dryperrein A	*D. perreticulata*	twigs and leaves	[136]
440	dryperrein B	*D. perreticulata*	twigs and leaves	[136]
441	dryperrein C	*D. perreticulata*	twigs and leaves	[136]
442	dryperrein D	*D. perreticulata*	twigs and leaves	[136]
443	(2*S*,3*R*,5*S*,10*R*)-2,3-dihydroxy-15,16-dinor-*ent*-pimar-8,11,13-triene	*F. fimbriata*	aerial parts	[39]
444	(2*S*,3*R*,5*S*,10*R*)-2-acetoxy-3-hydroxy-15,16-dinor-*ent*-pimar-8,11,13-triene	*F. fimbriata*	aerial parts	[39]
445	flickinflimilin B	*F. fimbriata*	leaves	[137]
446	flickinflimilin A	*F. fimbriata*	leaves	[137]
447	norflickinflimiod E	*F. fimbriata*	stems	[40]
448	norflickinflimiod F	*F. fimbriata*	stems	[40]
449	4*β*-carbometoxy-14-methyltotarol	*E. suaveolens*	stems	[138]
450	aspergiloid I	*Aspergillus* sp.	culture broth	[139]
451	aleuritin	*A. moluccanus*	twigs	[130]
452	salviprolin A	*S. przewalskii*	roots	[140]
453	salviprolin B	*S. przewalskii*	roots	[140]
454	commiphorane A	*R. Commiphora*	-	[141]
455	commiphorane B	*R. Commiphora*	-	[141]
456	commiphoranoid C	*R. Commiphora*	-	[142]
457	commiphorane K	*R. Commiphora*	-	[143]
458	7*β*-hydroxynagilactone D	*P. nagi*	seeds	[144]
459	3-*epi*-15-hydroxynagilactoneD	*P. nagi*	seeds	[144]
460	nagilactone K	*P. nagi*	seeds	[144]
461	nagilactone L	*P. nagi*	seeds	[144]
462	3*β*-hydroxynagilactone L	*P. nagi*	seeds	[144]
463	2*β*-hydroxynagilactone L	*P. nagi*	seeds	[144]
464	1*α*-chloro-2*β*,3*β*,15-trihydroxynagilactone	*P. nagi*	seeds	[144]
465	15-hydroxynagilactone L	*P. nagi*	seeds	[144]
466	cephanolide A	*C. sinensis*	-	[75,145]
467	cephanolide B	*C. sinensis*	-	[75,145]
468	cephanolide C	*C. sinensis*	-	[75,145]
469	sinuhirtone A	*S. hirta*	-	[118]

**Table 11:** Chemical constituents of C17 norditerpenes.

No.	Name	Plant Source	Plant Organ	Ref.
470	baccaramione A	*B. ramiflora*	twigs	[146]
471	baccaramione B	*B. ramiflora*	twigs	[146]
472	baccaramione C	*B. ramiflora*	twigs	[146]
473	baccaramione D	*B. ramiflora*	twigs	[146]
474	euphorane B	*E. dracunculoides*	whole plants	[52]
475	12-hydroxy-16,17-bis-*nor*-simonellite	*P. abrotanoides*	roots	[48]
476	nimbidiol	*P. abrotanoides*	roots	[48]
477	16,17-bis-nor-multicauline	*P. abrotanoides*	roots	[48]
478	salyunnanin D	*S. yunnanensis*	roots	[121]
479	salyunnanin E	*S. yunnanensis*	roots	[121]
480	(5*S*,8*R*,10*S*)-20-*nor*-militibetin A	*P. abrotanoides*	roots	[48]
481	sessilifol P	*C. sessilifolius*	-	[59]
482	sessilifol Q	*C. sessilifolius*	-	[59]
483	(5*S*,7*S*,10*R*)-7-deoxy-7,18-epoxynimbidiol	*C. sessilifolius*	-	[48]
484	dihydroneotanshinlactone	*S. digitaloides*	roots	[122]
485	neotanshinlactone	*S. digitaloides*	roots	[122]
486	danshenspiroketallactone	*S. digitaloides*	roots	[122]
487	rubesanolides F	*I. rubescens*	leaves	[57]
488	rubesanolide G	*I. rubescens*	leaves	[57]
489	pinuyunnanacid O	*P. yunnanensis*	resins	[147]
490	flueggrene A	*F. virosa*	roots	[148]
491	flueggrene B	*F. virosa*	roots	[148]
492	*epi*-6-oxonimbidiol	*C. angulatus*	root bark and leaves	[149]
493	(+)-7-deoxynimbidiol	*C. sessilifolius*	-	[150]
494	celaphanol A	*C. orbiculatus*	-	[150,151]
495	angulatusphenol C	*C. angulatus*	root bark and leaves	[149]
496	angulatusphenol D	*C. angulatus*	root bark and leaves	[149]
497	angulatusphenol E	*C. angulatus*	root bark and leaves	[149]
498	△^5^-nimbidiol	*C. angulatus*	root bark and leaves	[149]
499	margosolone	*C. angulatus*	root bark and leaves	[149]
500	demethylnimbionol	*C. angulatus*	root bark and leaves	[149]
501	13,15-dihydroxypodocarpa-8,11,13-triene	*P. banksiana* Lamb	buds	[152]
502	(3*R*,5*S*,9*R*,10*S*)-3-hydroxy-*ent*-podocarpa-8(14)-ene-13-one	*C. sessilifolius*	-	[153]
503	(4*R*,5*R*,9*R*,10*R*,13*S*)-13-hydroxypodocarp-8(14)-en-19-oic acid	*A. macrophylla*	-	[6]
504	(4*R*,5*R*,9*R*,10*R*,13*R*)-13-hydroxypodocarp-8(14)-en-19-oic acid	*A. macrophylla*	-	[6]
505	13-oxo-podocarp-8(14)-en-19-oic acid	*A. macrophylla*	-	[6]
506	angulatusphenol A	*C. angulatus*	root bark and leaves	[149]
507	angulatusphenol B	*C. angulatus*	root bark and leaves	[149]
508	7′,8′-*threo*-guaiacylglycerol-*α*,*γ*-*O*-nimbidiol diether	*C. sessilifolius*	-	[150]
509	(M)-bicelaphanol A	*C. orbiculatus*	root bark	[151,154]
510	(P)-bicelaphanol A	*C. orbiculatus*	root bark	[151,154]
511	angulatusdiphenol A	*C. angulatus*	root bark and leaves	[149]
512	trolliusditerpenoside N	*T. chinensis*	flowers	[155]
513	enbepeanone A	*A. grandiflflorum*	-	[156]
514	caesalminaxin M	*C. minax*	seeds	[157]
515	populusone	*P. euphratica*	exudates	[158]
516	pharicusin B	*I. pharicus*	aerial parts	[159]
517	(±)-8,13-secoepicavernosine	*Cacospongia* sp.	-	[160]
518	(+)-8,13-secocavernosine	*Cacospongia* sp.	-	[160]
519	(−)-8,13-secocavernosine	*Cacospongia* sp.	-	[160]
520	(+)-cavernosine	*Cacospongia* sp.	-	[160]
521	(−)-cavernosine	*Cacospongia* sp.	-	[160]

**Table 12:** Chemical constituents of C16 norditerpenes.

No.	Name	Plant Source	Plant Organ	Ref.
522	acrostalic acid	*P. sinensis*	needles and twigs	[132,161]
523	LL-Z1271-*β*	*A. wentii* EN-48	-	[161,162]
524	13,14,15,16-tetranorlabda-8(17)-en-12-carboxylic acid	*E. verrucosus*	-	[4]
525	3*α*-hydroxy-8*α*-acetoxy-13,14,15,16-tetranorlabdan-12-oeic acid	*S. aethiopis*	aerial parts	[163]
526	avxanthin A	*A. villosum*	rhizomes	[164]
527	elettarin A	*E. cardamomum*	fruits	[165]
528	elettarin B	*E. cardamomum*	fruits	[165]
529	asperolide C	*A. wentii* EN-48	-	[162]
530	botryosphaerin B	*P. sinensis*	needles and twigs	[162]
531	asperolide A	*A. wentii* EN-48	-	[162,166]
532	asperolide B	*A. wentii* EN-48	-	[162,166]
533	asperolide E	*A. wentii* EN-48	culture extract	[166]
534	tetranorditerpenoid derivative	*A. wentii* EN-48	-	[162]
535	wentilactone A	*A. wentii* EN-48	-	[162]
536	wentilactone B	*A. wentii* EN-48	culture extract	[162]
537	13,14,15,16-tetranorlabd-7-en-19,6*β*:12,17-diolide	*P. sinensis*	needles and twigs	[161]
538	botryosphaerin G	*P. sinensis*	needles and twigs	[161]
539	botryosphaerin H	*P. sinensis*	needles and twigs	[161]
540	3*α*,10*β*-dimethyl-1,2,3,3a,5a,7,10b,10c-octahydro-5,8-dioxa-acephenanthrylene-4,9-dione	*P. sinensis*	needles and twigs	[161]
541	botryosphaerin A	*P. sinensis*	needles and twigs	[161]
542	1-naphthaleneacetic-7-oxo-1,2,3,4,4a,7,8,8a-octahydro1,2,4a,5-tetramethyl acid	*P. longifolia*	leaves	[167]
543	methyl-7-oxo-1,2,3,4,4a,7,8,8a-octahydro-1,2,4a,5-tetramethyl-1-naph-thaleneacetate	*P. longifolia*	leaves	[167]
544	castanol C	*S. castanea*	flowers	[50]
545	sinubatin A	*S. nanolobata*	-	[168]
546	norcrocrassinone	*C. crassifolius*	roots	[169]
547	norcrassin A	*C. crassifolius*	roots	[170]
548	(3a*R*,5*S*,5a*R*,6*R*,9a*S*,9b*R*)-methyl5-hydroxy-3*α*,6,9*α*-trimethyl-2oxododecahydronaphtho[21-b]furan-6-carboxylate	*S. sahendica*	leaves	[171]
549	acerolanin A	*M. emarginata*	aerial parts	[172]
550	acerolanin B	*M. emarginata*	aerial parts	[172]
551	acerolanin C	*M. emarginata*	aerial parts	[172]
552	trigonochinene E	*T. flavidus*	stems	[173]
553	vibsanolide F	*V. odoratissimum*	leaves	[174]
554	vibsanolide G	*V. odoratissimum*	leaves	[174]
555	trigoflavidol A	*T. flavidus*	stems	[173]
556	trigoflavidol B	*T. flavidus*	stems	[173]
557	neoboutomannin	*T. flavidus*	stems	[173]

**Table 13:** Cytotoxic activity of norditerpenes.

Name	Extraction Solvent	Cancer Types	Cancer Cells	Activities (IC_50_)	Ref.
tinocapillin A (**38**)	95% EtOH	liver cancer cervical cancer	HepG-2 Hela	9.9 ± 1.5 μM 9.7 ± 3.4 μM	[20]
tinocallone C (**40**)	95% EtOH	lung cancer	A549	14.0 ± 0.9 μM	[20]
tinocapillin B (**41**)	95% EtOH	lung cancer liver cancer cervical cancer	A549 HepG-2 Hela	9.6 ± 1.2 μM 10.1 ± 1.1 μM 12.0 ± 1.0 μM	[20]
icacinlactone F (**76**)	80% aqueous MeOH	breast cancer	MDA-MB-435 MDA-MB-231	6.16 μM 8.94 μM	[28]
asperether A (**101**)	EtOAc	breast duct cancer	T-47D	10 μM	[38]
asperether B (**102**)	EtOAc	breast cancer liver cancer	MCF-7 SMMC-7721	14 μM 12 μM	[38]
cryptotanshinone (**148**)	80% EtOH	colon cancer gastric cancer lung cancer	HCT-8 BGC-823 A549	3.9 μM 8.3 μM 2.6 μM	[44]
euphorane C (**139**)	95% EtOH	liver cancer	HepG-2	6.95 μM	[52]
methyltanshinoate (**150**)	acetone	liver cancer lung cancer breast cancer colon cancer	SMMC-7721 A549 MCF-7 SW-480	4.07 μM 5.26 μM 3.44 μM 6.35 μM	[50]
amentotaxin C (**174**)	MeOH	cervical cancer lung cancer breast cancer ovarian cancer liver cancer colon cancer	HeLa A549 MDA-MB-231 SKOV3 Huh-7 HCT-116	5.1 μM 9.8 μM 6.8 μM 2.9 μM 4.1 μM 1.9 μM	[65]
20-oxohainanolidol (**205**)	95% EtOH	leukemia lung cancer	HL-60 A549	0.77 ± 0.05 μM 1.129 ± 0.057 μM	[77]
cephinoid H (**208**)	MeOH	lung cancer cervical cancer gastric cancer	A549 HeLa SGC-7901	0.10 μM 0.13 μM 0.14 μM	[78]
10-hydroxyharringtonolide (**223**)	-	lung cancer oral epidermoid cancer leukemia colon cancer	A549 KB HL-60 HT-29	3.683 ± 0.947 μM 2.325 ± 0.040 μM 1.038 ± 0.002 μM 2.108 ± 0.108 μM	[79]
6-en-harringtonolide (**229**)	-	lung cancer oral epidermoid cancer leukemia colon cancer	A549 KB HL-60 HT-29	7.804 ± 3.797 μM 5.115 ± 0.148 μM 2.319 ± 0.247 μM 4.890 ± 0.622 μM	[79]
tanshinone I (**393**)	acetone	leukemia lung cancer breast cancer pancreatic cancer	HL-60 A549 SK-BR-3 PANC-1	3.19 μM 6.64 μM 3.40 μM 3.67 μM	[56,122]
dihydroisotanshinone I (**394**)	80% EtOH	lung cancer	A549	2.7 μM	[44]
dihydrotanshinone (**395**)	acetone	leukemia liver cancer lung cancer breast cancer pancreatic cancer	HL-60 SMMC-7721 A549 SK-BR-3 PANC-1	2.36 μM 3.03 μM 5.15 μM 3.97 μM 2.75 μM	[122]
3*β*-hydroxymakilactone A (424)	acetone	gastric cancer breast cancer liver cancer pancreatic cancer	AGS MDA-MB-231 HepG-2 PANC-1	0.88 ± 0.01 μM 5.46 ± 1.12 μM 5.56 ± 1.73 μM 1.35 ± 0.08 μM	[131]
2*β*-hydroxymakilactone A (425)	MeOH	cervical cancer gastric cancer breast cancer	Hela AGS MDA-MB-231	0.87 ± 0.04 μM 0.38 ± 0.03 μM 4.23 ± 2.06 μM	[131]
inumakilactone A (**426**)	MeOH	cervical cancer gastric cancer breast cancer	Hela AGS MDA-MB-231	1.77 ± 0.69 μM 1.33 ± 0.05 μM 2.98 ± 1.06 μM	[131]
inumakilactone B (**428**)	MeOH	cervical cancer gastric cancer breast cancer liver cancer pancreatic cancer	Hela AGS MDA-MB-231 HepG-2 PANC-1	0.62 ± 0.18 μM 0.55 ± 0.21 μM 0.66 ± 0.15 μM 3.54 ± 1.45 μM 8.51 ± 2.65 μM	[131]
dryperrein C (**441**)	95% EtOH	lung cancer leukemia	A549 HL-60	8.50 μM 1.95 μM	[136]
dryperrein D (**442**)	95% EtOH	leukemia	HL-60	1.37 μM	[136]
salyunnanin E (**479**)	acetone	cervical cancer	HeLa	0.86 μM	[121]
neotanshinlactone (**485**)	acetone	breast cancer	SK-BR-3	4.07 μM	[122]

**Table 14:** Anti-inflammatory activity of norditerpenes.

Name	Pathways	Activities (IC_50_)	Ref.
callinteger B (**31**)	Inhibited IL-1*β* secretion and maturations of caspase-1 in a dose-dependent manner	9.9 ± 1.5 μM	[15]
18(4→14), 19(4→8)-bis-abeo-*nor*-isopimarane-1,5-diene-3-yl-3*β*-methoxy propyl pentanoate (**82**)	Inhibited of pro-inflammatory cyclooxygenases (COX-2, COX-1) and 5-lipoxygenase (5-LOX) enzymes	0.75 mg/mL	[30]
6-((*E*)-12-(furan-13-yl)-10-methylpent-10-en-9-yl)-6,7,8,8atetrahydro-3*H*-isochromen-1-(5*H*)-one (**279**)	Inhibited of 5-LOX enzymes	0.92 mg/mL	[89]
(2*S*,3*R*,5*S*,9*S*,10*S*,13*S*)-2-*O*-*E*-cinnamoyl-3-hydroxy-16-*nor*-*ent*-pimar-8(14)-en-15-oic acid (**110**)	Inhibited the NF-κB pathway in LPS-stimulated RAW264.7 cells	14.7 ± 1.8 μM	[39]
cephalotanin A (**190**)	Evaluated in an NF-kB pathway luciferase assay for inhibitory effects	4.12 ± 0.61 µM	[73]
salvialba acid (**163**)	Lowered the levels of ICAM-1 and VCAM-1 in HAECs induced by TNF-α	20 µM caused significant reductions in cell viability; 0.05, 0.5, 5, and 10 µM did not affect cell viability	[60]
cephinoid H (**208**)	Inhibited TNF-α-induced NF-κB activation	0.10 μM	[78]
5-*epi*-sinuleptolide (**255**)	Activated ARE expression and inhibited NO production and NF-κB expression in RAW264.7 macrophage cells	5.6 ± 0.2 µM 57.9 ± 0.4 µM 28.6 ± 0.2 µM	[82,86]
sinuleptolide (**261**)	Activated ARE expression and inhibited NO production and NF-κB expression in RAW264.7 macrophage cells	3.6 ± 0.3 µM 56.0 ± 0.3 µM 25.1 ± 0.5 µM	[82,86]
fragilolide A (**269**)	Activated ARE expression and inhibited NO production and NF-κB expression in RAW264.7 macrophage cells	1.2 ± 0.2 µM 27.8 ± 0.6 µM 12.5 ± 0.2 µM	[82,86]
celaphanol A (**494**)	Demonstrated moderate inhibitory activities against NF-κB activation in RAW264.7 macrophages	15 μM	[149,150,151]
angulatusphenol C (**495**)	Demonstrated moderate inhibitory activities against NF-κB activation in RAW264.7 macrophages	25 μM	[149,150,151]
angulatusphenol D (**496**)	Demonstrated moderate inhibitory activities against NF-κB activation in RAW264.7 macrophages	19 μM	[149,150,151]
demethylnimbionol (**500**)	Demonstrated moderate inhibitory activities against NF-κB activation in RAW264.7 macrophages	4 μM	[149,150,151]
15-*nor*-14-oxolabda-8(17),13(16)-dien-19-oic acid (**8**)	Inhibited LPS-induced nitric oxide (NO) production. Attenuated the expression of iNOS and COX-2 at both mRNA and protein levels by inhibiting the LPS-induced degradation of I-κBα and the activation of NF-κB, as well as reducing ERK phosphorylation	3.56 μM	[7]
ebractenoid A (**324**)	Inhibited the production of NO in LPS-induced macrophages	7.50 μM	[113]
ebractenoid B (**325**)	Inhibited the production of NO in LPS-induced macrophages	6.49 μM	[113]
hedychin F (**437**)	Inhibited the production of NO in LPS-induced macrophages	21.0 μM	[12]
flickinflimilin B (**445**)	Inhibited the production of NO and TNF-α in LPS-induced macrophages	<25.0 μM	[40,137]
flickinflimilin A (**446**)	Inhibited the production of NO and TNF-α in LPS-induced macrophages	<25.0 μM	[40,137]
norflickinflimiod E (**447**)	Inhibited the production of NO and TNF-α in LPS-induced macrophages	<25.0 μM	[40,137]
norflickinflimiod F (**448**)	Inhibited the production of NO and TNF-α in LPS-induced macrophages	<25.0 μM	[40,137]
przewalskin (**373**)	Inhibited iNOS expression in J774A.1 macrophages stimulated with LPS	-	[48]
(5*S*,6*S*,7*R*,10*R*)-16,17-bis-*nor*-6*β*-hydroxy-18,7-epoxyferruginol (**374**)	Inhibited iNOS expression in J774A.1 macrophages stimulated with LPS	-	[48]
(5*S*,10*R*)-16,17-bis-*nor*-pisiferanol (**378**)	Inhibited iNOS expression in J774A.1 macrophages stimulated with LPS	-	[48]
(5*S*,8*R*,10*S*)-20-*nor*-militibetin A (**480**)	Inhibited iNOS expression in J774A.1 macrophages stimulated with LPS	-	[48]
(+)-7-deoxynimbidiol (**493**)	Inhibited LPS-stimulated NO releases and pro-inflammatory mediators and suppressed iNOS and COX-2 expressions to prevent NO production	4.9 μM	[139]
7′,8′-*threo*-guaiacylglycerol-*α*,*γ*-*O*-nimbidiol diether (**508**)	Inhibited LPS-stimulated NO releases and pro-inflammatory mediators, suppressed iNOS and COX-2 expressions to prevent NO production	12.6 μM	[139]
13-*epi*-scabrolide C (**263**)	Inhibited the production of IL-12 and IL-6 in LPS-stimulated BMDCs	5.30 ± 0.21 μM 13.12 ± 0.64 μM	[82]
scrodentoid H (**307**)	Reduced LPS-induced inflammation and inhibited the JNK/STAT3 pathway in macrophages	-	[105]
scrodentoid I (**308**)	Reduced LPS-induced inflammation and inhibited the JNK/STAT3 pathway in macrophages	-	[105]
sinusiaetone A (**316**)	Inhibited LPS-induced inflammation in BV-2 microglia at a concentration of 20 μM and decreased the mRNA levels of pro-inflammatory cytokines IL-6 and IL-1*β*	-	[109]

## Data Availability

Source databases for these publications include the Science Citation Index (SCI), SCI Expanded.

## Abbreviations

A549	Human non-small cell lung cancer cells
AGS	Human gastric adenocarcinoma cells
ARE	Antioxidant response element
BGC-823	Human gastric adenocarcinoma cells
BMDCs	Bone marrow-derived cells
COXs	Cyclooxygenases
Con A	Knife-beetle protein A
ED_50_	Median effective dose
GlmU	GlcNAc-P uridyltransferase
HepG2	Human hepatocellular carcinoma cells
Hela	Human cervical carcinoma cells
Huh-7	Human hepatocellular carcinoma cells
HL-60	Human promyelocytic leukemia cells
HT-29	Human colon cancer cells
HCTs	Human colon cancer cells
HAECs	Human aortic endothelial cells
iNOS	Inducible nitric oxide synthase
IC_50_	50% inhibiting concentration
ICAM-1	Intercellular adhesion molecule-1
ILs	Interleukins
JNK	c-Jun NH_2_-terminal kinase
KBs	Human oral epidermoid carcinoma cells
LPS	Lipopolysaccharide
MIC	Minimum inhibitory concentration
MCF-7	Human breast cancer cells
MDA-MB	Triple-negative breast cancer cells
MG132	A proteasome inhibitor
NF-κB	Nuclear factor kappa B
NO	Nitric oxide
NGF	Nerve growth factor
PANC-1	Human pancreatic cancer cells
PDTC	Pyrrolidinedithiocarbamate
SMMC-7721	Human hepatocarcinoma cells
SW-480	Human colon cancer cells
SGC-7901	Human stomach cancer cells
SK-BR-3	Human breast cancer cells
SKOV3	Human ovarian cancer cells
STAT	Signal transducers and activators of transcription
T-47D	Human breast duct cancer cells
TNF-*α*	Tumor necrosis factor-*α*
TCM	Traditional Chinese medicine
VCAM-1	Vascular cell adhesion molecule-1

## References

[1] Shen Y., Liang W.J., Shi Y.N., Kennelly E.J., Zhao D.K. (2020). Structural diversity, bioactivities, and biosynthesis of natural diterpenoid alkaloids. Nat. Prod. Rep..

[2] Bian X.Q., Bai J., Hu X.L., Wu X., Xue C.M., Han A.H., Su G.Y., Hua H.M., Pei Y.H. (2015). Penioxalicin, a novel 3-nor-2,3-seco-labdane type diterpene from the fungus *Penicillium oxalicum* TW01-1. Tetrahedron Lett..

[3] Li Y.L., Liu W., Han S.Y., Zhang J., Xu W., Li Q., Cheng Z.B. (2020). Penitholabene, a rare 19-nor labdane-type diterpenoid from the deep-sea-derived fungus *Penicillium thomii* YPGA3. Fitoterapia.

[4] Yang Y.L., Yang X.Y., Zhang X.K., Song Z.T., Liu F., Liang Y., Zhang J., Jin D.Q., Xu J., Lee D. (2019). Bioactive terpenoids from *Euonymus verrucosus* var. pauciflorus showing no inhibitory activities. Bioorg. Chem..

[5] Zhang M., Linuma M., Wang J.S., Oyama M., Ito T., Kong L.Y. (2012). Terpenoids from *Chloranthus serratus* and their anti-inflammatory activities. J. Nat. Prod..

[6] Li Y., Wang T.T., Zhao J., Shi X., Hu S.C., Gao K. (2012). Norditerpenoids from *Agathis macrophylla*. Food Chem..

[7] Kim T.H., Li H., Wu Q., Lee H.J., Ryu J.H. (2013). A new labdane diterpenoid with anti-inflammatory activity from *Thuja orientalis*. J. Ethnopharmacol..

[8] Balbinot R.B., Oliveira J.A.M.D., Bernardi D.I., Melo U.Z., Zanqueta E.B., Endo E.H., Ribeiro F.M., Volpato H., Figueiredo M.C., Back D.F. (2019). Structural characterization and biological evaluation of 18-nor-ent-labdane diterpenoids from *Grazielia gaudichaudeana*. Chem. Biodivers..

[9] Yoshinori S., Sachie M., Suyatno S., Motoo T. (2011). Nine new norlabdane diterpenoids from the leaves of *Austroeupatorium inulifolium*. Helv. Chim. Acta.

[10] Chacon-Morales P.A., Amaro-Luis J.M., Fermin L.B.R., Peixoto P.A., Deffieux D., Pouysegu L., Quideau S. (2019). Preparation and bactericidal activity of oxidation derivatives of austroeupatol, an ent-nor-furano diterpenoid of the labdane series from *Austroeupatorium inulifolium*. Phytochem. Lett..

[11] Yin H., Luo J.G., Shan S.M., Wang X.B., Luo J., Yang M.H., Kong L.Y. (2013). Amomaxins A and B, two unprecedented rearranged labdane norditerpenoids with a nine-membered ring from *Amomum maximum*. Org. Lett..

[12] Zhao Q., Xiao L.G., Bi L.S., Si Y., Zhang X.M., Chen J.H., Liu H.Y. (2022). Hedychins E and F: Labdane-type norditerpenoids with anti-Inflammatory activity from the rhizomes of *Hedychium forrestii*. Org. Lett..

[13] Wang L.N., Zhang J.Z., Li X., Wang X.N., Xie C.F., Zhou J.C., Lou H.X. (2012). Pallambins A and B, unprecedented hexacyclic 19-*nor*-secolabdane diterpenoids from the Chinese liverwort *Pallavicinia ambigua*. Org. Lett..

[14] Wang Y.M., Jiang W.Q., Feng Q., Lu H., Zhou Y.P., Liao J., Wang Q.T., Sheng G.Y. (2017). Identification of 15-nor-cleroda-3,12-diene in a Dominican amber. Org. Geochem..

[15] Bi D.W., Xiong F., Cheng B., Zhou Y.L., Zeb M.A., Tang P., Pang W.H., Zhang R.H., Li X.L., Zhang X.J. (2022). Callintegers A and B, unusual tricyclo[4.4.0.0^9,10^]tetradecane clerodane diterpenoids from *Callicarpa integerrima* with inhibitory effects on NLRP3 inflammasome activation. J. Nat. Prod..

[16] Soares B.A., Firme C.L., Maciel M.A.M., Kaiser C.R., Schilling E., Bortoluzzi A.J. (2014). Experimental and NMR theoretical methodology applied to geometric analysis of the bioactive clerodane trans-dehydrocrotonin. J. Braz. Chem. Soc..

[17] Zou M.F., Pan Y.H., Hu R., Yuan F.Y., Huang D., Tang G.H., Li W., Yin S. (2021). Highly modified *nor*-clerodane diterpenoids from *Croton yanhuii*. Fitoterapia.

[18] Pan Z.H., Ning D.S., Wu X.D., Huang S.S., Li D.P., Lv S.H. (2015). New clerodane diterpenoids from the twigs and leaves of *Croton euryphyllus*. Bioorg. Med. Chem. Lett..

[19] Zou M.F., Hu R., Liu Y.X., Fan R.Z., Xie X.L., Yin S. (2020). Two highly oxygenated nor-clerodane diterpenoids from *Croton caudatus*. J. Asian. Nat. Prod. Res..

[20] Wang B., Zhang P.L., Zhou M.X., Shen T., Zou Y.X., Lou H.X., Wang X.N. (2016). New *nor*-clerodane-type furanoditerpenoids from the rhizomes of *Tinospora capillipes*. Phytochem. Lett..

[21] Ye G.H., Xue J.J., Liang W.L., Yang S.J. (2021). Three new bioactive diterpenoids from the roots of *Croton crassifolius*. Nat. Prod. Res..

[22] Lv H.W., Luo J.G., Zhu M.D., Shan S.M., Kong L.Y. (2014). Teucvisins A-E, five new *neo*-clerodane diterpenes from *Teucrium viscidum*. Chem. Pharm. Bull..

[23] Huang D., Luo X.K., Yin Z.Y., Xu J., Gu Q. (2020). Diterpenoids from the aerial parts of *Flueggea acicularis* and their activity against RANKL-induced osteoclastogenesis. Bioorg. Chem..

[24] Yang L., Guo P.Y., Liu A.J., Sui S.Y., Shi S., Guo S.X., Dai J.G. (2019). Aquilariaenes A–H, eight new diterpenoids from *Chinese eaglewood*. Fitoterapia.

[25] Shin B., Kim B.Y., Cho E.J., Oh K.B., Shin J.H., Goodfellow M., Oh D.C. (2016). Actinomadurol, an antibacterial norditerpenoid from a rare actinomycete, *Actinomadura* sp. KC 191. J. Nat. Prod..

[26] Guo B., Zhao M., Wu Z.L., Onakpa M.M., Burdette J.E., Che C.T. (2020). 19-nor-pimaranes from *Icacina trichantha*. Fitoterapia.

[27] Xu M.M., Zhou J.F., Zeng L.P., Xu J.C., Onakpa M.M., Duan J.A., Che C.T., Bi H.K., Zhao M. (2021). Pimarane-derived diterpenoids with anti-Helicobacter pylori activity from the tuber of *Icacina trichantha*. Org. Chem. Front..

[28] Zhao M., Onakpa M.M., Chen W.L., Santarsiero B.D., Swanson S.M., Burdette J.E., Asuzu I.U., Che C.T. (2015). 17-Norpimaranes and (9βH)-17-norpimaranes from the tuber of *Icacina trichantha*. J. Nat. Prod..

[29] Li C.X., Li B., Ye J., Zhang W.D., Shen Y.H., Yin J. (2013). A new norditerpenoid from *Euonymus grandiflorus* Wall. Nat. Prod. Res..

[30] Joy M., Chakraborty K. (2017). An unprecedented antioxidative isopimarane norditerpenoid from bivalve clam, *Paphia malabarica* with anti-cyclooxygenase and lipoxygenase potential. Pharm. Biol..

[31] Wang X.N., Bashyal B.P., Wijeratne E.M.K., U'Ren J.M., Liu M.P., Gunatilaka M.K., Arnold A.E., Gunatilaka A.A.L. (2011). Smardaesidins A-G, isopimarane and 20-nor-isopimarane diterpenoids from *Smardaea* sp., a fungal endophyte of the moss *Ceratodon purpureus*. J. Nat. Prod..

[32] Di Lecce R., Masi M., Linaldeddu B.T., Pescitelli G., Maddau L., Evidente A. (2020). Bioactive specialized metabolites produced by the emerging pathogen *Diplodia olivarum*. Beilstein. Archi..

[33] Kato H., Sebe M., Nagaki M., Eguchi K., Kagiyama I., Hitora Y., Frisvad J.C., Williams R.M., Tsukamoto S. (2019). Taichunins A-D, Norditerpenes from *Aspergillus taichungensis*. J. Nat. Prod..

[34] Li X.D., Li X.M., Li X., Xu G.M., Liu Y., Wang B.G. (2016). Aspewentins D–H, 20-nor-isopimarane derivatives from the deep sea sediment-derived fungus *Aspergillus wentii* SD-310. J. Nat. Prod..

[35] Li X.D., Lin X., Li X.M., Xu G.M., Liu Y., Wang B.G. (2018). 20-nor-isopimarane epimers produced by *Aspergillus wentii* SD-310, a fungal strain obtained from deep sea sediment. Mar. Drugs.

[36] Miao F.P., Liang X.R., Liu X.H., Ji N.Y. (2014). Aspewentins A–C, norditerpenes from a cryptic pathway in an algicolous strain of *Aspergillus wentii*. J. Nat. Prod..

[37] Chen H.P., Li J., Zhao Z.Z., Li X.Y., Liu S.L., Wang Q.Y., Liu J.K. (2020). Diterpenes with bicyclo[2.2.2]octane moieties from the fungicolous fungus *Xylaria longipes* HFG1018. Org. Biomol. Chem..

[38] Li X., Li X.M., Li X.D., Xu G.M., Liu Y., Wang B.G. (2016). 20-Nor-isopimarane cycloethers from the deep-sea sediment-derived fungus *Aspergillus wentii* SD-310. RSC Adv..

[39] Li H., Zhao J.J., Chen J.L., Zhu L.P., Wang D.M., Jiang L., Yao D.P., Zhao Z.M. (2015). Diterpenoids from aerial parts of *Flickingeria fimbriata* and their nuclear factor-kappaB inhibitory activities. Phytochemistry.

[40] Chen J.L., Zhao Z.M., Xue X., Tang G.H., Zhu L.P., Yang D.P., Jiang L. (2014). Bioactive norditerpenoids from *Flickingeria fimbriata*. RSC Adv..

[41] Li C.J., Dai W.F., Liu D., Jiang M.Y., Zhang Z.J., Chen X.Q., Chen C.H., Li R.T., Li H.M. (2020). Bioactive ent-isopimarane diterpenoids from *Euphorbia neriifolia*. Phytochemistry.

[42] Wang P.X., Xie C.F., An L.J., Yang X.Y., Xi Y.R., Yuan S., Zhang C.Y., Tuerhong M., Jin D.Q., Lee D.H. (2019). Bioactive diterpenoids from the stems of *Euphorbia royleana*. J. Nat. Prod..

[43] Kadir A., Zheng G.J., Zheng X.F., Jin P.F., Maiwulanjiang M., Gao B., Aisa H.A., Yao G.M. (2021). Structurally diverse diterpenoids from the roots of *Salvia deserta* based on nine different skeletal types. J. Nat. Prod..

[44] Zhang D.W., Liu X., Xie D., Chen R.D., Tao X.Y., Zou J.H., Dai J.G. (2013). Two new diterpenoids from cell cultures of *Salvia miltiorrhiza*. Chem. Pharm. Bull..

[45] Kang J., Li L., Wang D.D., Wang H.Q., Liu C., Li B.M., Yan Y., Fang L.H., Du G.H., Chen R.Y. (2015). Isolation and bioactivity of diterpenoids from the roots of *Salvia grandifolia*. Phytochemistry.

[46] Hussain A., Adhikari A., Choudhary M.I., Ayatollahi S.A., Atta-Ur-Rahman (2016). New adduct of abietane-type diterpene from *Salvia leriifolia* Benth. Nat. Prod. Res..

[47] Eghtesadi F., Farimani M.M., Hazeri N., Valizadeh J. (2016). Abietane and nor-abitane diterpenoids from the roots of *Salvia rhytidea*. SpringerPlus.

[48] Alizadeh Z., Farimani M.M., Parisi V., Marzocco S., Ebrahimi S.N., Tommasi N.D. (2021). Nor-abietane diterpenoids from *Perovskia abrotanoides* roots with anti-inflammatory potential. J. Nat. Prod..

[49] Cao Q., Wang S.X., Chen Y.X. (2019). Abietane diterpenoids with potent cytotoxic activities from the resins of *Populus euphratica*. Nat. Prod. Commune.

[50] Pan Z.H., Li Y., Wu X.D., He J., Chen X.Q., Xu G., Peng L.Y., Zhao Q.S. (2012). Norditerpenoids from *Salvia castanea* Diels f. pubescens. Fitoterapia.

[51] Li J.Y., Peng Y., Li L.Z., Gao P.Y., Gao C., Xia S.X., Song S.J. (2013). Two new abietane diterpenoids from the roots of *Tripterygium wilfordii* Hook. f. Helv. Chim. Acta.

[52] Yan X.L., Zou M.F., Chen B.L., Yuan F.Y., Zhu Q.F., Zhang X., Lin Y., Long Q.D., Liu W.L., Liao S.G. (2022). Euphorane C, an unusual C17-norabietane diterpenoid from *Euphorbia dracunculoides* induces cell cycle arrest and apoptosis in human leukemia K562 cells. Arab. J. Chem..

[53] Zhao H.M., Li H.L., Huang G.L., Chen Y.G. (2017). A new abietane mono-norditerpenoid from *Podocarpus nagi*. Nat. Prod. Res..

[54] Lusarczyk S., Senol Deniz F.S., Abel R., Pecio Ł., Pérez-Sánchez H., Cerón-Carrasco J.P., Den-Haan H., Banerjee P., Preissner R., Krzyżak E. (2020). Norditerpenoids with selective anti-cholinesterase activity from the roots of *Perovskia atriplicifolia* Benth. Int. J. Mol. Sci..

[55] Jiang H.L., Wang X.Z., Xiao J., Luo X.H., Yao X.J., Zhao Y.Y., Chen Y.J., Crews P., Wu Q.X. (2013). New abietane diterpenoids from the roots of *Salvia przewalskii*. Tetrahedron.

[56] Cheng Q.Q., He Y.F., Li G., Liu Y.J., Gao W., Huang L.Q. (2013). Effects of combined Eelicitors on tanshinone metabolic profiling and SmCPS expression in *Salvia miltiorrhiza* hairy root culture. Molecules.

[57] He K., Zou J., Wang Y.X., Zhao C.L., Ye J.H., Zhang J.J., Pan L.T., Zhang H.J. (2021). Rubesanolides F and G: Two novel lactone-type norditerpenoids from *Isodon rubescens*. Molecules.

[58] Yu P., Zhang S.D., Li Y.L., Yang X.W., Zeng H.W., Li H.L., Zhang W.D. (2012). Abieseconordines A and B, two novel norditerpenoids with a 18-nor-5,10: 9,10-disecoabietane skeleton from *Abies forrestii*. Helv. Chim. Acta.

[59] Wang L.J., Xiong J., Liu S.T., Pan L.L., Hu J.F. (2015). ent-Abietane-type and related seco-/nor-diterpenoids from the rare chloranthaceae plant *Chloranthus sessilifolius* and their nntineuroinflammatory activities. J. Nat. Prod..

[60] Xie T.T., Ma S.L., Lou H.X., Zhu R.X., Sun L.R. (2014). Two novel abietane norditerpenoids with anti-inflammatory properties from the roots of *Salvia miltiorrhiza* var. alba. Tetrahedron Lett..

[61] Wang W.G., Yan B.C., Li X.N., Du X., Wu H.Y., Zhan R., Li Y., Pu J.X., Sun H.D. (2014). 6,7-Seco-ent-kaurane-type diterpenoids from *Isodon eriocalyx* var. laxiflora. Tetrahedron.

[62] Zou J., Du X., Pang G., Shi Y.M., Wang W.G., Zhan R., Kong L.M., Li X.N., Li Y., Pu J.X. (2012). Ternifolide A, a new diterpenoid possessing a rare macrolide motif from *Isodon ternifolius*. Org. Lett..

[63] Ueno A.K., Barcellos A.F., Costa-Silva T.A., Mesquita J.T., Ferreira D.D., Tempone A.G., Romoff P., Antar G.M., Lago J.H.G. (2018). Antitrypanosomal activity and evaluation of the mechanism of action of diterpenes from aerial parts of *Baccharis retusa*. Fitoterapia.

[64] Faiella L., Piaz F.D., Bader A., Braca A. (2014). Diterpenes and phenolic compounds from *Sideritis pullulans*. Phytochemistry.

[65] Li H., Liang Y.R., Chen S.X., Wang W.X., Zou Y.K., Nuryyeva S., Houk K.N., Xiong J., Hu J.F. (2020). Amentotaxins C–V, Structurally diverse diterpenoids from the leaves and twigs of the vulnerable conifer *Amentotaxus argotaenia* and their cytotoxic effects. J. Nat. Prod..

[66] Kennedy M.L., Llanos G.G., Castanys S., Gamarro F., Bazzocchi I.L., Jimenez I.A. (2011). Terpenoids from *Maytenus* species and assessment of their reversal activity against a multidrug-resistant *Leishmania tropica* line. Chem. Biodivers..

[67] Ren H., Xu Q.L., Luo Y., Zhang M., Zhou Z.Y., Dong L.M., Tan J.W. (2015). Two new ent-kaurane diterpenoids from *Wedelia trilobata* (L.) *Hitchc*. Phytochem. Lett..

[68] Isyaka S.M., Mas-Claret E., Langat M.K., Hodges T., Selway B., Mbala B.M., Mvingu B.K., Mulholland D.A. (2020). Cytotoxic diterpenoids from the leaves and stem bark of *Croton haumanianus* (Euphorbiaceae). Phytochemistry.

[69] Zhou X.Q., Li S.Q., Liao C.C., Dai W.F., Rao K.R., Ma X.R., Li R.T., Chen X.Q. (2021). Structurally diversified ent-kaurane and abietane diterpenoids from the stems of *Tripterygium wilfordii* and their anti-inflammatory activity. Bioorg. Chem..

[70] Zhou C.X., Sun L.R., Feng F., Mo J.X., Zhu H., Yang B., He Q.J., Gan L.S. (2013). Cytotoxic diterpenoids from the stem bark of *Annona squamosa* L.. Helv. Chim. Acta.

[71] Yang J., Wang W.G., Wu H.Y., Liu M., Jiang H.Y., Du X., Li Y., Pu J.X., Sun H.D. (2017). ent-Kaurene diterpenoids from *Isodon phyllostachys*. Tetrahedron Lett..

[72] Zhan R., Li X.N., Du X., Wang W.G., Dong K., Su J., Li Y., Pu J.X., Sun H.D. (2013). Bioactive ent-kaurane diterpenoids from *Isodon rosthornii*. J. Nat. Prod..

[73] Xu J.B., Fan Y.Y., Gan L.S., Zhou Y.B., Li J., Yue J.M. (2016). Cephalotanins A–D, four norditerpenoids represent three highly rigid carbon skeletons from *Cephalotaxus sinensis*. Chemistry.

[74] Ge Z.P., Zhou B., Zimbres F.M., Cassera M.B., Zhao J.X., Yue J.M. (2022). Cephalotane-type norditerpenoids from *Cephalotaxus fortunei* var. alpine. Chin. J. Chem..

[75] Fan Y.Y., Xu J.B., Liu H.C., Gan L.S., Ding J., Yue J.M. (2017). Cephanolides A–J, cephalotane-type diterpenoids from *Cephalotaxus sinensis*. J. Nat. Prod..

[76] Ge Z.P., Liu H.C., Wang G.C., Liu Q.F., Xu C.H., Ding J., Fan Y.Y., Yue J.M. (2019). 17-*nor*-Cephalotane-type diterpenoids from *Cephalotaxus fortunei*. J. Nat. Prod..

[77] Zhao J.X., Fan Y.Y., Xu J.B., Gan L.S., Xu C.H., Ding J., Yue J.M. (2017). Diterpenoids and lignans from *Cephalotaxus fortune*. J. Nat. Prod..

[78] Ni L., Zhong X.H., Chen X.J., Zhang B.J., Bao M.F., Cai X.H. (2018). Bioactive norditerpenoids from *Cephalotaxus fortunei* var. alpina and C. lanceolata. Phytochemistry.

[79] Ni G., Zhang H., Fan Y., Liu H., Ding J., Yue J.M. (2016). Mannolides A–C with an intact diterpenoid skeleton providing insights on the biosynthesis of antitumor *Cephalotaxus* Troponoids. Org. Lett..

[80] Ge Z.P., Fan Y.Y., Deng W.D., Zheng C.Y., Li T., Yue J.M. (2021). Cephalodiones A-D: Compound characterization and semisynthesis by [6+6] cycloaddition. Angew. Chem. Int. Ed. Engl..

[81] Cui W.X., Yang M., Li H., Li S.W., Yao L.G., Li G., Tang W., Wang C.H., Liang L.F., Guo Y.W. (2020). Polycyclic furanobutenolide-derived norditerpenoids from the South China Sea soft corals *Sinularia scabra* and *Sinularia polydactyla* with immunosuppressive activity. Bioorg. Chem..

[82] Nguyen P.T., Nguyen H.N., Nguyen X.C., Quang T.H., Tung P.T., Dat L.D., Chae D., Kim S., Koh Y.S., Kiem P.V. (2013). Anti-inflammatory norditerpenoids from the soft coral *Sinularia maxima*. Bioorg. Med. Chem. Lett..

[83] Wang C.L., Jin T.Y., Liu X.H., Zhang J.R., Shi X., Wang M.F., Huang R.F., Zhang Y., Liu K.C., Li G.Q. (2022). Sinudenoids A–E, C19-norcembranoid diterpenes with unusual scaffolds from the soft coral *Sinularia densa*. Org. Lett..

[84] Craig R.A., Smith R.C., Roizen J.L., Jones A.C., Virgil S.C., Stoltz B.M. (2018). Development of a unified enantioselective, convergent synthetic Aapproach toward the furanobutenolide-derived polycyclic norcembranoid diterpenes: Asymmetric formation of the polycyclic norditerpenoid carbocyclic core by tandem annulation cascade. J. Org. Chem..

[85] Thomas S.A.L., von Salm J.L., Clark S., Ferlita S., Nemani P., Azhari A., Rice C.A., Wilson N.G., Kyle D.E., Baker B.J. (2018). Keikipukalides, furanocembrane diterpenes from the antarctic deep sea octocoral *Plumarella delicatissima*. J. Nat. Prod..

[86] Cheng W., Ji M., Li X.D., Ren J.W., Yin F.L., van Ofwegen L., Yu S.W., Chen X.G., Lin W.H. (2017). Fragilolides A–Q, norditerpenoid and briarane diterpenoids from the gorgonian coral *Junceella fragilis*. Tetrahedron.

[87] Liu J., Tang Q., Huang J., Li T., Ouyang H., Lin W.H., Yan X.J., Yan X., He S. (2022). Sinuscalide A: An antiviral norcembranoid with an 8/8-fused carbon scaffold from the South China Sea soft coral *Sinularia scabra*. J. Org. Chem..

[88] Liu W.W., Zhang Y., Yuan C.M., Yu C., Ding J.Y., Li X.X., Hao X.J., Wang Q., Li S.L. (2014). Japodagricanones A and B, novel diterpenoids from *Jatropha podagrica*. Fitoterapia.

[89] Chakraborty K., Krishnan S., Joy M. (2021). Antioxidative oxygenated terpenoids with bioactivities against pro-inflammatory inducible enzymes from Indian squid, *Uroteuthis* (Photololigo) *duvaucelii*. Nat. Prod. Res..

[90] Li L.L., Chen L., Li Y.H., Sun S.K., Ma S.G., Li Y., Qu J. (2020). Cassane and nor-cassane diterpenoids from the roots of *Erythrophleum fordii*. Phytochemistry.

[91] Kamikawa S., Oshimo S., Ohta E., Nehira T., Omura H., Ohta S. (2016). Cassane diterpenoids from the roots of *Caesalpinia decapetala* var. japonica and structure revision of caesaljapin. Phytochemistry.

[92] Sun P., Cao D.H., Xiao Y.D., Zhang Z.Y., Wang J.N., Shi X.C., Xiao C.F., Hu H.B., Xu Y.K. (2020). Aspidoptoids A–D: Four new diterpenoids from *Aspidopterys obcordata* vine. Molecules.

[93] Zhao J.Q., Lv J.J., Wang Y.M., Xu M., Zhu H.T., Wang D., Yang C.R., Wang Y.F., Zhang Y.J. (2013). Phyllanflexoid C: First example of phenylacetylene-bearing 18-nor-diterpenoid glycoside from the roots of *Phyllanthus flexuosus*. Tetrahedron Lett..

[94] Li F.L., Lin S., Zhang S.T., Pan L.F., Chai C.W., Su J.C., Yang B.Y., Liu J.J., Wang J.P., Hu Z.X. (2020). Modified fusicoccane-type diterpenoids from *Alternaria brassicicola*. J. Nat. Prod..

[95] Wu J., Zhang H., He L.M., Xue Y.Q., Jia J., Wang S.B., Zhu K.K., Hong K., Cai Y.S. (2021). A new fusicoccane-type norditerpene and a new indone from the marine-derived fungus *Aspergillus aculeatinus* WHUF0198. Chem. Biodivers..

[96] Bie Q., Chen C.M., Yu M.Y., Guo J.R., Wang J.P., Liu J.J., Zhou Y., Zhu H.C., Zhang Y.H. (2019). Dongtingnoids A–G: Fusicoccane diterpenoids from a *Penicillium* Species. J. Nat. Prod..

[97] Kawakami S., Toyoda H., Harinantenaina L., Matsunami K., Otsuka H., Shinzato T., Takeda Y., Kawahata M., Yamaguchi K. (2013). Eight new diterpenoids and two new nor-diterpenoids from the stems of *Croton cascarilloides*. Chem. Pharm. Bull..

[98] Liang W.J., Geng C.A., Zhang X.M., Chen H., Yang C.Y., Rong G.Q., Zhao Y., Xu H.B., Wang H., Zhou N.J. (2014). (±)-Paeoveitol, a pair of new norditerpene enantiomers from *Paeonia veitchii*. Org. Lett..

[99] Zhao J.X., Liu C.P., Qi W.Y., Han M.L., Han Y.S., Wainberg M.A., Yue J.M. (2014). Eurifoloids A–R, structurally diverse diterpenoids from *Euphorbia neriifolia*. J. Nat. Prod..

[100] Oanh V.T.K., Ha N.T.T., Duc H.V., Thuc D.N., Hang N.T.M., Thanh L.N. (2021). New triterpene and nor-diterpene derivatives from the leaves of *Adinandra poilanei*. Phytochem. Lett..

[101] Liang X.R., Miao F.P., Song Y.P., Liu X.H., Ji N.Y. (2016). Citrinovirin with a new norditerpene skeleton from the marine algicolous fungus *Trichoderma citrinoviride*. Bioorg. Med. Chem. Lett..

[102] White A.M., Pierens G.K., Forster L.C., Winters A.E., Cheney K.L., Garson M.J. (2016). Rearranged diterpenes and norditerpenes from three Australian Goniobranchus Mollusks. J. Nat. Prod..

[103] Han G.Y., Sun D.Y., Liang L.F., Yao L.G., Chen K.X., Guo Y.W. (2018). Spongian diterpenes from Chinese marine sponge *Spongia officinalis*. Fitoterapia.

[104] Zhang L.Q., Zhao Y.Y., Huang C., Chen K.X., Li Y.M. (2016). Scrodentoids F-I, four C_19_-norditerpenoids from *Scrophularia dentate*. Tetrahedron.

[105] Mao G.H., Sun L.Q., Xu J.W., Li Y.M., Dunzhu C., Zhang L.Q., Qian F. (2020). Scrodentoids H and I, a pair of natural epimerides from *Scrophularia dentata*, inhibit inflammation through JNK-STAT3 axis in THP-1 cells. Evid. Based Complement. Alternat. Med..

[106] San-Martin A., Bacho M., Nunez S., Rovirosa J., Soler A., Blanc V., Leon R., Olea A.F. (2018). A novel normulinane isolated from *Azorella compacta* and assessment of its antibacterial activity. J. Chil. Chem. Soc..

[107] Lam Y.T.H., Palfner G., Lima C., Porzel A., Brandt W., Frolov A., Sultani H., Franke K., Wagner C., Merzweiler K. (2019). Nor-guanacastepene pigments from the Chilean mushroom *Cortinarius pyromyxa*. Phytochemistry.

[108] Nidhal N., Zhou X.M., Chen G.Y., Zhang B., Han C.R., Song X.P. (2020). Chemical constituents of *Leucas zeylanica* and their chemotaxonomic significance. Biochem. Syst. Ecol..

[109] Chen Z.H., Li W.S., Zhang Z.Y., Luo H., Wang J.R., Zhang H.Y., Zeng Z.R., Chen B., Li X.W., Guo Y.W. (2021). Sinusiaetone A, an anti-inflammatory norditerpenoid with a bicyclo[11.3.0]hexadecane nucleus from the Hainan soft coral *Sinularia siaesensis*. Org. Lett..

[110] Wang S.S., Cheng Y.B., Lin Y.C., Liaw C.C., Chang J.Y., Kuo Y.H., Shen Y.C. (2015). Nitrogen-containing diterpenoids, sesquiterpenoids, and *nor*-diterpenoids from *Cespitularia taeniata*. Mar. Drugs.

[111] Lin Y.C., Lin C.C., Chu Y.C., Fu C.W., Sheu J.H. (2021). Bioactive diterpenes, norditerpenes, and sesquiterpenes from a Formosan soft coral *Cespitularia* sp.. Pharmaceuticals.

[112] Gao P.Y., Li L.Z., Liu K.C., Sun C., Sun X., Wu Y.N., Song S.J. (2017). Natural terpenoid glycosides with in vitro/vivo antithrombotic profiles from the leaves of *Crataegus pinnatifida*. RSC Adv..

[113] Liu Z.G., Li Z.L., Bai J., Meng D.L., Li N., Pei Y.H., Zhao F., Hua H.M. (2014). Anti-inflammatory diterpenoids from the roots of *Euphorbia ebracteolata*. J. Nat. Prod..

[114] Zhang Y., Liu Y.B., Li Y., Li L., Ma S.G., Qu J., Jiang J.D., Chen X.G., Zhang D., Yu S.S. (2015). Terpenoids from the roots of *Alangium chinense*. J. Asian Nat. Prod. Res..

[115] Li L.Z., Liang X., Sun X., Qi X.L., Wang J., Zhao Q.C., Song S.J. (2016). Bioactive norditerpenoids and neolignans from the roots of *Salvia miltiorrhiza*. Org. Biomol. Chem..

[116] Rusman Y., Wilson M.B., Williams J.M., Held B.W., Blanchette R.A., Anderson B.N., Lupfer C.R., Salomon C.E. (2020). Antifungal norditerpene oidiolactones from the fungus *Oidiodendron truncatum*, a potential biocontrol agent for whitenose syndrome in bats. J. Nat. Prod..

[117] Olivon F., Retailleau P., Desrat S., Touboul D., Roussi F., Apel C., Litaudon M. (2020). Isolation of picrotoxanes from *Austrobuxus carunculatus* using taxonomy-based molecular networking. J. Nat. Prod..

[118] Olivon F., Retailleau P., Desrat S., Touboul D., Roussi F., Apel C., Litaudon M. (2022). Sinuhirtone A, an uncommon 17, 19-dinorxeniaphyllanoid, and nine related new terpenoids from the Hainan soft coral *Sinularia hirta*. Mar. Drugs.

[119] Prieto I.M., Paola A., Perez M., Garcia M., Blustein G., Schejter L., Palermo J.A. (2022). Antifouling Diterpenoids from the Sponge *Dendrilla Antarctica*. Chem. Biodivers..

[120] Wei Y.L., Wang C., Cheng Z.B., Tian X.G., Jia J.M., Cui Y.L., Feng L., Sun C.P., Zhang B.J., Ma X.C. (2017). Diterpenoids isolated from *Euphorbia ebracteolata* roots and their inhibitory effects on α-Glucosidase. J. Nat. Prod..

[121] Wu C.Y., Liao Y., Yang Z.G., Yang X.W., Shen X.L., Li R.T., Xu G. (2014). Cytotoxic diterpenoids from *Salvia yunnanensis*. Phytochemistry.

[122] Xu G., Yang J., Wang Y.Y., Peng L.Y., Yang X.W., Pan Z.H., Liu E.D., Li Y., Zhao Q.S. (2010). Diterpenoid constituents of the roots of *Salvia digitaloides*. J. Agric. Food Chem..

[123] Yao F., Zhang D.W., Qu G.W., Li G.S., Dai S.J. (2012). New abietane norditerpenoid from *Salvia miltiorrhiza* with cytotoxic activities. J. Asian. Nat. Prod. Res..

[124] Zhu Q., Tang C.P., Ke C.Q., Li X.Q., Liu J., Gan L.S., Weiss H., Gesing E., Ye Y. (2010). Constituents of *Trigonostemon chinensis*. J. Nat. Prod..

[125] Jiang Z.Y., Zhou J., Huang C.G., Hu Q.F., Huang X.Z., Wang W., Zhang L.Z., Li G.P., Xia F.T. (2015). Two novel antiviral terpenoids from the cultured *Perovskia atriplicifolia*. Tetrahedron.

[126] Zhu Q., Tang C.P., Mandi A., Kurtan T., Ye Y. (2020). Trigonostemons G and H, dinorditerpenoid dimers with axially chiral biaryl linkage from *Trigonostemon chinensis*. Chirality.

[127] Wang X.F., Liu F.F., Zhu Z.D., Fang Q.Q., Qu S.J., Zhu W.L., Yang L., Zuo J.P., Tan C.H. (2019). Flueggenoids A–E, new dinorditerpenoids from *Flueggea virosa*. Fitoterapia.

[128] Chao C.H., Cheng J.C., Shen D.Y., Wu T.S. (2014). Anti-hepatitis C virus dinorditerpenes from the roots of *Flueggea virosa*. J. Nat. Prod..

[129] Zhu J.Y., Zhang C.Y., Dai J.J., Rahman K., Zhang H. (2017). Diterpenoids with thioredoxin reductase inhibitory activities from *Jatropha multifida*. Nat. Prod. Res..

[130] Chang F.R., Wang S.W., Chen S.R., Lee C.Y., Sheu J.H., Cheng Y.B. (2020). Aleuritin, a novel dinor-diterpenoid from the twigs of *Aleurites moluccanus* with an anti-lymphangiogenic effect. Org. Biomol. Chem..

[131] Qi Y.Y., Su J., Zhang Z.J., Li L.W., Fan M., Zhu Y., Wu X.D., Zhao Q.S. (2018). Two new anti-proliferative C_18_-norditerpenes from the roots of *Podocarpus macrophyllus*. Chem. Biodivers..

[132] Huang T., Ying S.H., Li J.Y., Chen H.W., Zang Y., Wang W.X., Li J., Xiong J., Hu J.F. (2020). Phytochemical and biological studies on rare and endangered plants endemic to China. Part XV. Structurally diverse diterpenoids and sesquiterpenoids from the vulnerable conifer *Pseudotsuga sinensis*. Phytochemistry.

[133] Mirzania F., Farimani M.M., Sarrafi Y., Ebrahimi S.N., Troppmair J., Kwiatkowski M., Stuppner H., Alilou M. (2021). New sesterterpenoids from *Salvia mirzayanii* Rech.f. and Esfand. stereochemical characterization by computational electronic circular dichroism. Front. Chem..

[134] Cheng B., Fang F., Zhou Q.T., Li Y., Wu X.W., Zhao X.R., Bi D.W., Zhang X.J., Zhang R.H., Ji X. (2023). Highly oxygenated labdane diterpenoids from *Stevia rebaudiana* and their anti-atherosclerosis activities. Chem. Biodivers..

[135] Lv X.J., Li Y., Ma S.G., Qu J., Liu Y.B., Li L., Wang R.B., Yu S.S. (2017). Isopimarane and nor-diterpene glucosides from the twigs and leaves of *Lyonia ovalifolia*. Tetrahedron.

[136] Ge Y.Z., Zhang H., Liu H.C., Dong L., Ding J., Yue J.M. (2014). Cytotoxic dinorditerpenoids from *Drypetes perreticulata*. Phytochemistry.

[137] Chen J.L., Zhong W.J., Tang G.H., Li J., Zhao Z.M., Yang D.P., Jiang L. (2014). Norditerpenoids from *Flickingeria fimbriata* and their inhibitory activities on nitric oxide and tumor necrosis factor-α production in mouse macrophages. Molecules.

[138] Dade J.M.E., Kablan L.A., Okpekon T.A., Say M., Yapo K.D., Komlaga G., Boti J.B., Koffi A.P., Guei L.E., Djakoure L.A. (2015). Cassane diterpenoids from stem bark of *Erythrophleum suaveolens*. Phytochem. Lett..

[139] Guo Z.K., Wang R., Huang W., Li X.N., Jiang R., Tan R.X., Ge H.M. (2014). Aspergiloid I, an unprecedented spirolactone norditerpenoid from the plant-derived endophytic fungus *Aspergillus* sp. YXf3. Beilstein J. Org. Chem..

[140] Su X.D., Wu Y.C., Wu M.F., Lu J.F., Jia S.J., He X., Liu S.N., Zhou Y.Y., Xing H., Xue Y.B. (2021). Regioisomers Salviprolin A and B, unprecedented rosmarinic acid conjugated dinorditerpenoids from *Salvia przewalskii* Maxim. Molecules.

[141] Dong L., Cheng L.Z., Yan Y.M., Wang S.M., Cheng Y.X. (2017). Commiphoranes A-D, carbon skeletal terpenoids from *Resina commiphora*. Org. Lett..

[142] Zhu S.S., Liu J.W., Yan Y.M., Liu Y., Mao Z., Cheng Y.X. (2020). Terpenoids from *Resina Commiphora* regulating lipid metabolism via activating PPARα and CPT1 expression. Org. Lett..

[143] Yang X.H., Wang D.W., Yan Y.M., Jiao Y.B., Cheng Y.X., Wang F. (2021). Commiphoranes K-O, new terpenoids from *Resina Commiphora* and their anti-inflammatory activities. Chem. Biodivers..

[144] Feng Z.L., Zhang L.L., Zheng Y.D., Liu Q.Y., Liu J.X., Feng L., Huang L., Zhang Q.W., Lu J.J., Lin L.G. (2017). Norditerpenoids and dinorditerpenoids from the seeds of *Podocarpus nagi* as cytotoxic agents and autophagy inducers. J. Nat. Prod..

[145] Zhang H.Y., He H.B., Gao S.H. (2021). Asymmetric total synthesis of cephanolide B. Org. Chem. Front..

[146] Chen S.S., Tong X., Liu X.Y., Zheng C.Y., Zhou J.S., Fan Y.Y., He S.J., Zhou B., Yue J.M. (2023). Baccaramiones A–D, four highly oxygenated and rearranged trinorditerpenoids from *Baccaurea ramiflora*. J. Org. Chem..

[147] Liu Y.F., Yang B.C., Song Z.M., Qiao L.Q., Peng R., Feng W.S., Cheng Y.X., Wang Y.Z. (2023). Seven diterpenoids from the resin of *Pinus yunnanensis* Franch and their anti-inflammatory activity. Fitoterapia.

[148] Chao C.H., Cheng J.C., Hwang T.L., Shen D.Y., Wu T.S. (2014). Trinorditerpenes from the roots of *Flueggea virosa*. Bioorg. Med. Chem. Lett..

[149] Wang J.X., Jin H., Li H.L., Zhang W.D. (2018). Podocarpane trinorditerpenes from *Celastrus angulatus* and their biological activities. Fitoterapia.

[150] Jang H.J., Kim K.H., Park E.J., Kang J.A., Yun B.S., Lee S.J., Park C.S., Lee S., Lee S.W., Rho M.C. (2020). Anti-inflammatory activity of diterpenoids from *Celastrus orbiculatus* in lipopolysaccharide-stimulated RAW264.7 cells. J. Immunol. Res..

[151] Wang L.Y., Wu J., Yang Z., Wang X.J., Fu Y., Liu S.Z., Wang H.M., Zhu W.L., Zhang H.Y., Zhao W.M. (2013). (M)- and (P)-Bicelaphanol A, dimeric trinorditerpenes with promising neuroprotective activity from *Celastrus orbiculatus*. J. Nat. Prod..

[152] Georges P., Legault J., Lavoie S., Grenon C., Pichette A. (2012). Diterpenoids from the buds of *Pinus banksiana* Lamb. Molecules.

[153] Wang L.J., Xiong J., Lau C., Pan L.L., Hu J.F. (2015). Sesquiterpenoids and further diterpenoids from the rare Chloranthaceae plant *Chloranthus sessilifolius*. J. Asian Nat. Prod. Res..

[154] Wang X.J., Wang L.Y., Fu Y., Wu J., Tang X.C., Zhao W.M., Zhang H.Y. (2013). Promising effects on ameliorating mitochondrial function and enhancing Akt signaling in SH-SY5Y cells by (M)-bicelaphanol A, a novel dimeric podocarpane type. Phytomedicine.

[155] Guo M.L., Xu H.T., Yang J.J., Chou G.X. (2021). Diterpenoid glycosides from the flower of *Trollius chinensis* Bunge and their nitric oxide inhibitory activities. Bioorg. Chem..

[156] Thomas S.A.L., Sanchez A., Kee Y., Wilson N.G., Baker B.J. (2019). Bathyptilones: Terpenoids from an antarctic sea pen, *Anthoptilum grandiflorum* (Verrill, 1879). Mar. Drugs.

[157] Zheng Y., Zhang S.W., Xuan L.J. (2015). Trinorcassane and cassane diterpenoids from the seeds of *Caesalpinia minax*. Fitoterapia.

[158] Liu K.X., Zhu Y.X., Yan Y.M., Zeng Y., Jiao Y.B., Qin F.Y., Liu J.W., Zhang Y.Y., Cheng Y.X. (2019). Discovery of populusone, a skeletal stimulator of umbilical cord mesenchymal stem cells from *Populus euphratica* exudates. Org. Lett..

[159] Hu Z.X., Xu H.C., Hu K., Liu M., Li X.N., Li X.R., Du X., Zhang Y.H., Puno P., Sun H.D. (2018). Structurally diverse diterpenoids from *Isodon pharicus*. Org. Chem. Front..

[160] Zhang X.W., Li P.L., Qin G.F., Li S.Y., de Voogd N.J., Tang X.L., Li G.Q. (2019). Isolation and absolute configurations of diversiform C_17_, C_21_ and C_25_ terpenoids from the marine sponge *Cacospongia* sp.. Mar. Drugs.

[161] Chen Y.M., Yang Y.H., Li X.N., Zou C., Zhao P.J. (2015). Diterpenoids from the endophytic fungus *Botryosphaeria* sp. P483 of the Chinese herbal medicine *Huperzia serrate*. Molecules.

[162] Sun H.F., Li X.M., Meng L., Cui C.M., Gao S.S., Li C.S., Huang C.G., Wang B.G. (2012). Asperolides A–C, tetranorlabdane diterpenoids from the marine alga-derived endophytic fungus *Aspergillus wentii* EN-48. J. Nat. Prod..

[163] Srivedavyasasri R., White M.B., Kustova T.S., Gemejiyeva N.G., Cantrell C.L., Ross S.A. (2018). New tetranorlabdanoic acid from aerial parts of *Salvia aethiopis*. Nat. Prod. Res..

[164] Yin H., Dan W.J., Fan B.Y., Guo C., Wu K., Li D., Xian K.F., Pescitelli G., Gao J. (2019). M Anti-inflammatory and α-glucosidase inhibitory activities of labdane and norlabdane diterpenoids from the rhizomes of *Amomum villosum*. J. Nat. Prod..

[165] Liang S., Luo J.G., Wang Z., Wang X.B., Kong L. (2017). New tetranorlabdane diterpenoids from the fruits of *Elettaria cardamomum* Maton. Phytochem. Lett..

[166] Li X.D., Li X., Li X.M., Xu G.M., Zhang P., Meng L.H., Wang B.G. (2016). Tetranorlabdane diterpenoids from the deep sea sediment-derived fungus *Aspergillus wentii* SD-310. Planta Med..

[167] Afolabi S., Olorundare O., Ninomiya M., Babatunde A., Mukhtar H., Koketsu M. (2017). Comparative antileukemic activity of a tetranorditerpene isolated from *Polyalthia longifolia* leaves and the derivative against human leukemia HL-60 cells. J. Oleo Sci..

[168] Hsu F.Y., Wang S.K., Duh C.Y. (2018). Xeniaphyllane-derived terpenoids from soft coral *Sinularia nanolobata*. Mar. Drugs.

[169] Zhang Z.X., Li H.H., Zhi D.J., Wu P.Q., Hu Q.L., Yu Y.F., Zhao Y., Yu C.X., Fei D.Q. (2018). Norcrocrassinone: A novel tetranorditerpenoid possessing a 6/6/5 fused ring system from *Croton crassifolius*. Tetrahedron Lett..

[170] Zhang Z.X., Wu P.Q., Li H.H., Qi F.M., Fei D.Q., Hu Q.L., Liu Y.H., Huang X.L. (2018). Norcrassin A, a novel C_16_ tetranorditerpenoid, and bicrotonol A, an unusual dimeric labdane-type diterpenoid, from the roots of *Croton crassifolius*. Org. Biomol. Chem..

[171] Mofidi Tabatabaei S., Salehi P., Moridi Farimani M., Neuburger M., De Mieri M., Hamburger M., Nejad-Ebrahimi S. (2017). A nor-diterpene from *Salvia sahendica* leaves. Nat. Prod. Res..

[172] Liu J.Q., Deng Y.Y., Li T.Z., Han Q., Li Y., Qiu M.H. (2014). Three new tetranorditerpenes from aerial parts of acerola cherry (*Malpighia emarginata*). Molecules.

[173] Tang G.H., Zhang Y., Gu Y.C., Li S.F., Di Y.T., Wang Y.H., Yang C.X., Zuo G.Y., Li S.L., He H.P. (2012). Trigoflavidols A–C, degraded diterpenoids with antimicrobial activity, from *Trigonostemon flavidus*. J. Nat. Prod..

[174] Li S.F., Yu X.Q., Li Y.L., Bai M., Lin B., Yao G.D., Song S.J. (2021). Vibsane-type diterpenoids from *Viburnum odoratissimum* and their cytotoxic activities. Bioorg. Chem..

